# Assessing the impact of vaccination and behavioural change on Mpox transmission in high-risk groups in the Democratic Republic of Congo using an age-structured mathematical model

**DOI:** 10.1016/j.onehlt.2026.101324

**Published:** 2026-01-14

**Authors:** Andrew Omame, Nicola Luigi Bragazzi, Ali Asgary, Chigozie Louisa J. Ugwu, Jude Dzevela Kong, Jianhong Wu, Woldegebriel Assefa Woldegerima

**Affiliations:** a Disease-Informed Modelling, Methods, and Systems (DIMMS) Laboratory, Department of Mathematics and Statistics, York University, Toronto, Ontario, Canada; b Laboratory for Industrial and Applied Mathematics (LIAM), Department of Mathematics and Statistics, York University, 4700 Keele Street, Toronto, M3J 1P3, Ontario, Canada; c Department of Clinical Pharmacy, Saarland University, 66123, Saarbrücken, Germany; d School of Administrative Studies, Faculty of Liberal Arts and Professional Studies, York University, 4700 Keele Street, Toronto, M3J 1P3, Ontario, Canada; e Africa-Canada Artificial Intelligence and Data Innovation Consortium (ACADIC), Canada; f Artificial Intelligence & Mathematical Modeling Lab (AIMM Lab), Dalla Lana School of Public Health, University of Toronto, 155 College St Room 500, Toronto, ON M5T 3M7, Canada; g Department of Mathematics, Federal University of Technology, Owerri, Imo State, Nigeria; h Department of Statistics, Faculty of Physical Sciences, University of Nigeria, Nsukka, Enugu State, Nigeria; i Department of Mathematics, CNCS, Mekelle University, Tigray, Ethiopia

**Keywords:** Mpox, Vaccination, Age-structure, DRC, High-risk, Low-risk, Control reproduction number, Modelling

## Abstract

Mpox is a viral zoonotic disease that has gained global attention due to its recurrent outbreaks in endemic regions of Africa and beyond. The recent clade I outbreak in the Democratic Republic of the Congo (DRC) has been characterized by extensive transmission among children – particularly those under 15 years of age – and adults with elevated occupational risks, such as healthcare workers, sex workers, and hunters. Motivated by emerging evidence that vaccination alone may not explain the observed decline in mpox transmission across the DRC, and recognizing that behavioural modification is more feasible among adults, this study investigates the synergistic impact of vaccination and behaviour-driven contact reduction among high-risk adults within an age- and risk-structured modelling framework. The model stratifies the population into adults (high- and low-risk groups) and children. It incorporates vaccination for both adults and children, as well as behavioural adaptations (in the form of contact reduction) among high-risk adults. The model is calibrated to weekly reported mpox cases in the DRC from January 2024 to April 2025, from which key parameters are estimated. Scenario analyses reveal that among the adult population, behavioural change has a greater impact than vaccination in reducing mpox transmission. The model indicated that vaccination targeting children yielded the most significant effects, in comparison to either contact-reduction measures or immunization of adults. Moreover, our results indicate that initiating a 50% reduction in contact rates among high-risk adults approximately 20 weeks earlier yields an additional 20% decrease in the cumulative number of mpox cases, compared with implementing the same reduction concurrently with the vaccination intervention in the DRC. Given the current low vaccination coverage and supply constraints, our findings provide evidence-based guidance for optimizing vaccine allocation and prioritizing behavioural interventions among high-risk groups to prevent sustained transmission.

## 1 Introduction

The mpox virus, formerly referred to as monkeypox, is a member of the Orthopoxvirus genus, which also comprises notable pathogens such as the variola virus, vaccinia virus and cowpox virus [1]. Mpox is a large, double-stranded DNA virus, and thus exhibits greater genomic complexity relative to many RNA viruses, including those responsible for influenza and COVID-19. The virus was first identified in 1958 during investigations involving captive monkeys [2], although current evidence strongly implicates rodents as the principal reservoir, particularly within Central and West African regions [3]. Human infection was first documented in the Democratic Republic of the Congo (DRC) in the early 1970s, and subsequent outbreaks have occurred intermittently, predominantly across endemic regions in African but with increasing global incidence [4].

**Fig. 1:**
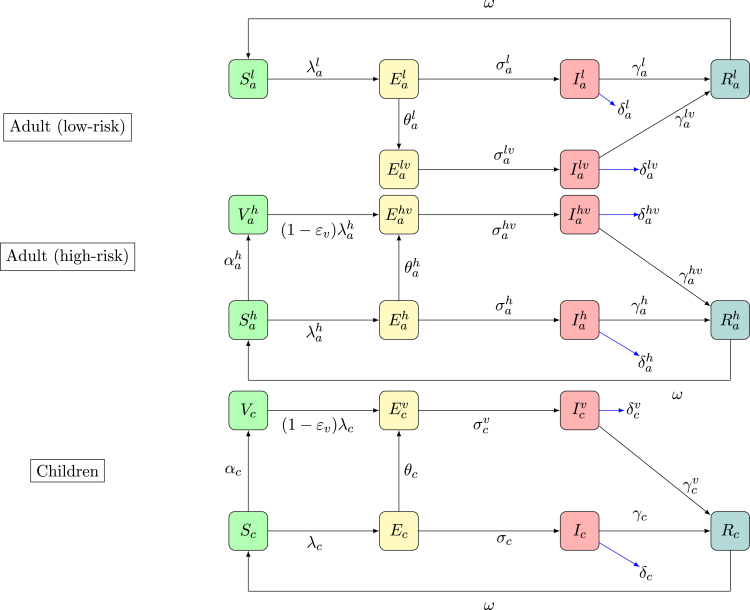
Schematic diagram of the model. Solid black lines show the transitions between the compartments.

The epidemiological characteristics of the recent mpox outbreak in the DRC and nearby countries in Africa showed marked differences from those observed in previous and the recent 2022 global clade IIb outbreaks [12], [13]. In the ongoing outbreak in the DRC, more than half of reported cases occurred in children below the age of 15, an age group that also bears the highest mortality burden [14], [15]. Recent investigations have reported clusters linked to sexual contacts [16], [17]. While detailed data partitioned according to occupation remain limited, available records shows high case clusters among sex workers [18]. Furthermore, case fatality rates are substantially higher in this setting, with estimates of approximately 5% among adults and up to 10% among children. Clinical manifestations frequently include disseminated skin rashes or genital lesions, and there is growing evidence of adverse pregnancy outcomes, including pregnancy loss, among infected women [19], [20]. The extensive case profiles of the recent mpox clade I outbreak among children [21] (especially, those below the age of 15 years) and adults with occupational risks (particularly sex workers and miners) [22], [23], [24] point to a mixed transmission route, with infections occurring through both regular community and sexual contacts. In particular, according to World Health Organization (WHO), about 67% of all reported mpox cases in the DRC in 2024 occurred among children below the age of 15 years, with about 30% of the cases attributed to adults in the high-risk group (predominantly among sex workers and miners) [23], [25], [26]. The WHO has categorized the high-risk groups for mpox (based on exposure) as health-care workers, people in the same household or close community as someone who has contracted mpox, including children; individuals with multiple sexual partners and sex workers of any gender and their clients [9].

**Table 1:** Model parameters.

Parameter	Description	Value	Reference

$t_{a}$	Time at which intervention started in the DRC	October 5, 2024	[5]
$\beta_{a}^{s}\left(t\right)$	Time-dependent sexual transmission rate among adults in high-risk group	$\left\{\begin{array}{c} 0.07149,\;t\leq t_{a},\;\\ 0.03437,\;t>t_{a}\; \end{array}\right)$	Fitted
$\beta_{a}^{hh}\left(t\right)$	Time dependent non-sexual transmission rate among adults in high-risk group	$\left\{\begin{array}{c} 0.07142,\;t\leq t_{a},\;\\ 0.03425,\;t>t_{a}\; \end{array}\right)$	Fitted
$\beta_{a}^{hl}$	Transmission rate between susceptible adults in the high-risk group and infected adults low-risk groups	0.015 day^−1^	Fitted
$\beta_{a}^{lh}$	Transmission rate between susceptible adults in the low-risk group and infected adults high-risk groups	0.005094 day^−1^	Fitted
$\beta_{a}^{ll}$	Transmission rate among adults in the low-risk group	0.007378 day^−1^	Fitted
$\beta_{ac}^{h}$	Transmission rate between susceptible adults in the high-risk group and infected children	0.08966 day^−1^	Fitted
$\beta_{ac}^{l}$	Transmission rate between susceptible adults in the low-risk group and infected children	0.005178 day^−1^	Fitted
$\beta_{cc}$	Transmission rate among children	0.1764 day^−1^	Fitted
$\beta_{ca}$	Transmission rate between susceptible children and infected adults	0.09479 day^−1^	Fitted
${ɛ}_{v}$	Mpox vaccine efficacy against incident infection	0.85−0.95	[6], [7]
φ	Modification parameter for reduced infectiousness of vaccinated infected individuals	0.1−0.9	[8]
ω	Waning rate of infection-acquired immunity	$\frac{1}{112}$ day^−1^	[8]
$\alpha_{a}^{h}$	Pre-exposure vaccination rate for adults in the high-risk group	0.01−0.05	Assumed
$\alpha_{c}$	Pre-exposure vaccination rate for children	0.01−0.05	Assumed
$\gamma_{a}^{h},\gamma_{a}^{l},\gamma_{c}$	Mean infectious period for adults and children	$\frac{1}{14}$ day^−1^	[9]
$\sigma_{a}^{h},\sigma_{a}^{l},\sigma_{c}$	Mean incubation period for adults and children	$\frac{1}{8.5}$ day^−1^	[9], [10]
$\delta_{a}^{h}$	Mpox-induced death rate for adults in high-, low-risk group	0.005	Assumed
$\delta_{a}^{l}$	Mpox-induced death rate for adults in the low-risk group	0.005	Assumed
$\delta_{c}$	Mpox-induced death rate for children	0.005	Assumed
$\theta_{a}^{h}$	Post-exposure vaccination rate for adults in the high-risk group	0.01−0.05	Assumed
$\theta_{a}^{l}$	Post-exposure vaccination rate for adults in the low-risk group	Assumed	0.01−0.05
$\theta_{c}$	Post-exposure vaccination rate for children	0.01−0.05	Assumed
N(0)	Total population of DRC	105,044,646	[11]

In light of the ongoing mpox outbreak, the WHO has recommended vaccination (either as a pre-exposure prophylaxis or post-exposure prophylaxis) for individuals in the high-risk groups as one of the most effective interventions for preventing and controlling the spread of mpox [27], [28]. Some vaccines currently approved for use against mpox by the WHO include ACAM2000, the Modified Vaccinia Ankara Vaccine-Bavarian Nordic (MVA-BN) and LC16m8 (also known as LC16-KMB). These vaccines have proven to be highly effective in both preventing mpox infection and reducing severity. [6], [7], [29], [30], [31]. Despite the proven effectiveness of recommended vaccines, some recent studies have highlighted that vaccination alone may not fully describe the noticeable decline in mpox cases in the DRC [32], [33], [34], [35]. For instance, the authors [32] observed that, in addition to vaccination, behavioural adaptations played dominant role in reducing mpox spread in endemic settings. Consequently, targeting behavioural change specifically among high-risk adult populations while maintaining vaccination efforts for both adults and children offers a more realistic and synergistic approach to controlling the spread of mpox. Thus, our model assumes that behavioural change in response to the ongoing mpox outbreak in the DRC is concentrated among high-risk adults. We therefore adopt behaviour as a time-varying reduction in effective contact rates that is driven primarily by the awareness and behavioural response of the high-risk adult subpopulation. This parameterization both reflects field evidence of heterogeneity in risk perception and response and allows the model to capture the disproportionate effect that high-risk adult behavioural change exerts on transmission dynamics in mixed-risk, age-structured populations.

Mathematical models have been developed to understand the dynamics and control of mpox incorporating animal–human transmission [36], [37], [38], [39]. Some other models focused on the transmission patterns of the 2022 mpox outbreak within the high-risk or MSM population involving sexual transmission, vaccination and/or behavioural change [40], [41], [42], [43], [44], [45], [46], [47], [48], [49], [50], [51], [52]. Additionally, some studies have also been carried out to understand and recommend control measures for the recent 2023–2024 mpox outbreak and the situation in endemic countries using mathematical and statistical models [26], [53], [54]. However, the development of a comprehensive mathematical model to assess the combined impact of vaccination and behavioural change among high-risk groups in the DRC remains an area requiring further exploration.

The primary objective of this study is to assess the impact of vaccination and behavioural change on Mpox transmission in high-risk groups in the Democratic Republic of Congo using an age-structured mathematical model. The population is stratified into children and adults, with the adult group further subdivided into high- and low-risk groups defined by behavioural and occupational characteristics. The model incorporates pre-exposure vaccination for high-risk adults and children, as well as post-exposure vaccination across all age groups. To the best of the authors’ knowledge, this is the first detailed age- and risk-structured model designed to assess the combined impacts of vaccination and behavioural change on mpox transmission dynamics in the DRC.

## 2 Mathematical model formulation

We propose a compartmental model which based on the framework of susceptible–vaccinated–exposed–infectious–recovered (SVEIR). The model in this study comprises of 22 mutually exclusive compartments. The compartments are grouped into adults (further sub-categorized into high- and low-risk groups) and children. The adults compartments are as follows: $S_{a}^{h}$: unvaccinated susceptible adults (high-risk group), $V_{a}^{h}$: vaccinated adults (high-risk group) prone to infection, $V_{a}^{hb}$: vaccinated adults (high-risk group) who have developed immunity to infection due to pre-exposure vaccination, $E_{a}^{h}$: unvaccinated exposed adults (high-risk group), $E_{a}^{hv}$: vaccinated exposed adults (high-risk group), $I_{a}^{h}$: unvaccinated infectious adults (high-risk group), $I_{a}^{hv}$: vaccinated infectious adults (high-risk group), $R_{a}^{h}$: recovered adults (high-risk group), $S_{a}^{l}$: susceptible adults (low-risk group), $E_{a}^{l}$: unvaccinated exposed adults (low-risk group), $E_{a}^{lv}$: vaccinated exposed adults (low-risk group), $I_{a}^{l}$: unvaccinated infectious adults (low-risk group), $I_{a}^{lv}$: vaccinated infectious adults (low-risk group), $R_{a}^{l}$: recovered adults (low-risk group). The children compartments are sub-grouped as follows: $S_{c}$: unvaccinated susceptible children, $V_{c}$: vaccinated children prone to infection, $V_{c}^{b}$: vaccinated children who have developed immunity to infection due to pre-exposure vaccination, $E_{c}$: unvaccinated exposed children, $E_{c}^{v}$: vaccinated exposed children, $I_{c}$: unvaccinated infectious children, $I_{c}^{v}$: vaccinated infectious children, $R_{c}$: recovered children.

In our model, adults are defined as individuals aged 15 years and older, while children are those younger than 15 years. Consistent with WHO definitions, the high-risk adult group comprises individuals with occupational or living conditions that increase exposure risk (for example, sex workers and their clients, health-care workers, persons involved in animal trade, miners, and individuals residing in crowded settings such as internally displaced persons (IDP) camps) [22], [23], [24]. Low-risk adults are those who do not belong to the high-risk group but remain susceptible to mpox via community transmission. Although children are generally not engaged in high-risk occupations or behaviours, they have been reported to face substantial exposure risk to mpox Clade I through community transmission and to be at increased risk of developing complications. [55], [56], [57]. The model assumes that transmission among high-risk adults occurs via both sexual and non-sexual (community) contacts, whereas transmission within the low-risk group and between adults and children occurs primarily through community spread. For simplicity, sexual transmission in the model is restricted to heterosexual contacts and is assumed to occur mainly within the high-risk adult group. The schematic diagram of the model is shown in Fig. 1, and a detailed description of model variables and parameters is provided in Table 1.

The model equations are given as follows:

(2.1) $$\text{Adults (Low-risk)}\;\left\{\begin{array}{c} \frac{dS_{a}^{l}}{dt}=-\lambda_{a}^{l}S_{a}^{l}+\omega \;R_{a}^{l},\\ \frac{dE_{a}^{l}}{dt}=\lambda_{a}^{l}\;S_{a}^{l}-\left(\sigma_{a}^{l}+\theta_{a}^{l}\right)E_{a}^{l},\\ \frac{dE_{a}^{lv}}{dt}=\theta_{a}^{l}E_{a}^{l}-\sigma_{a}^{lv}E_{a}^{lv},\\ \frac{dI_{a}^{l}}{dt}=\sigma_{a}^{l}\;E_{a}^{l}-\left(\delta_{a}^{l}+\gamma_{a}^{l}\right)\;I_{a}^{l},\\ \frac{dI_{a}^{lv}}{dt}=\sigma_{a}^{lv}\;E_{a}^{lv}-\left(\delta_{a}^{lv}+\gamma_{a}^{lv}\right)\;I_{a}^{lv},\\ \frac{dR_{a}^{l}}{dt}=\gamma_{a}^{l}\;I_{a}^{l}+\gamma_{a}^{lv}I_{a}^{lv}-\omega \;R_{a}^{l}, \end{array}\right)$$

$$\text{Adults (High-risk)}\;\left\{\begin{array}{c} \frac{dS_{a}^{h}}{dt}=-\alpha_{a}^{h}S_{a}^{h}-\lambda_{a}^{h}S_{a}^{h}+\omega \;R_{a}^{h},\\ \frac{dV_{a}^{h}}{dt}=\alpha_{a}^{h}S_{a}^{h}-\left(1-{ɛ}_{v}\right)\;\lambda_{a}^{h}\;V_{a}^{h},\\ \frac{dE_{a}^{h}}{dt}=\lambda_{a}^{h}\;S_{a}^{h}-\left(\sigma_{a}^{h}+\theta_{a}^{h}\right)E_{a}^{h},\\ \frac{dE_{a}^{hv}}{dt}=\lambda_{a}^{h}\;\left(1-{ɛ}_{v}\right)V_{a}^{h}+\theta_{a}^{h}E_{a}^{h}-\sigma_{a}^{hv}E_{a}^{hv},\\ \frac{dI_{a}^{h}}{dt}=\sigma_{a}^{h}\;E_{a}^{h}-\left(\delta_{a}^{h}+\gamma_{a}^{h}\right)\;I_{a}^{h},\\ \frac{dI_{a}^{hv}}{dt}=\sigma_{a}^{hv}\;E_{a}^{hv}-\left(\delta_{a}^{hv}+\gamma_{a}^{hv}\right)\;I_{a}^{hv},\\ \frac{dR_{a}^{h}}{dt}=\gamma_{a}^{h}\;I_{a}^{h}+\gamma_{a}^{hv}I_{a}^{hv}-\omega \;R_{a}^{h}, \end{array}\right)$$

$$\text{Children}\;\left\{\begin{array}{c} \frac{dS_{c}}{dt}=-\alpha_{c}S_{c}-\lambda_{c}S_{c}+\omega \;R_{c},\\ \frac{dV_{c}}{dt}=\alpha_{c}S_{c}-\left(1-{ɛ}_{v}\right)\;\lambda_{c}\;V_{c},\\ \frac{dE_{c}}{dt}=\lambda_{c}\;S_{c}-\left(\sigma_{c}+\theta_{c}\right)E_{c},\\ \frac{dE_{c}^{v}}{dt}=\lambda_{c}\;\left(1-{ɛ}_{v}\right)V_{c}+\theta_{c}E_{c}-\sigma_{c}^{v}E_{c}^{v},\\ \frac{dI_{c}}{dt}=\sigma_{c}\;E_{c}-\left(\delta_{c}+\gamma_{c}\right)\;I_{c},\\ \frac{dI_{c}^{v}}{dt}=\sigma_{c}^{v}\;E_{c}^{v}-\left(\delta_{c}^{v}+\gamma_{c}^{v}\right)\;I_{c}^{v},\\ \frac{dR_{c}}{dt}=\gamma_{c}\;I_{c}+\gamma_{c}^{v}I_{c}^{v}-\omega \;R_{c}, \end{array}\right)$$

where, the forces of infection for mpox transmission among adults (in the high- and low-risk groups) and children are respectively given by:

(2.2) $$\lambda_{a}^{h}=\frac{\beta_{a}^{s}\left(I_{a}^{h}+\varphi I_{a}^{hv}\right)}{N_{a}^{h}}+\frac{\beta_{a}^{hh}\left(I_{a}^{h}+\varphi I_{a}^{hv}\right)}{N_{a}^{h}}+\frac{\beta_{a}^{hl}\left(I_{a}^{l}+\varphi I_{a}^{lv}\right)}{N_{a}^{l}}+\frac{\beta_{ac}^{h}\left(I_{c}+\varphi I_{c}^{v}\right)}{N_{c}}$$

$$\lambda_{a}^{l}=\frac{\beta_{a}^{lh}\left(I_{a}^{h}+\varphi I_{a}^{hv}\right)}{N_{a}^{h}}+\frac{\beta_{a}^{ll}\left(I_{a}^{l}+\varphi I_{a}^{lv}\right)}{N_{a}^{l}}+\frac{\beta_{ac}^{l}\left(I_{c}+\varphi I_{c}^{v}\right)}{N_{c}}$$

$$\lambda_{c}=\frac{\beta_{cc}\left(I_{c}+\varphi I_{c}^{v}\right)}{N_{c}}+\frac{\beta_{ca}\left(I_{a}^{h}+\varphi I_{a}^{hv}+I_{a}^{l}+\varphi I_{a}^{lv}\right)}{N_{a}^{h}+N_{a}^{l}}$$

where $\beta_{a}^{s}$ and $\beta_{a}^{hh}$ denote the sexual and non-sexual transmission rates within the high-risk adult group, respectively. The model also accounts for interactions between adults in the high- and low-risk groups: $\beta_{a}^{hl}$ is the transmission rate from infectious low-risk adults to susceptible high-risk adults, $\beta_{a}^{lh}$ is the transmission rate from infectious high-risk adults to susceptible low-risk adults, and $\beta_{a}^{ll}$ is the transmission rate within the low-risk adult group. Interactions between adults (both risk groups) and children are represented by $\beta_{ac}^{h}$ for transmission from infectious children to susceptible high-risk adults and $\beta_{ac}^{l}$ for transmission from infectious children to susceptible low-risk adults. The parameters $\beta_{cc}$ and $\beta_{ca}$ denote transmission among children and from infectious adults to susceptible children, respectively. The mpox vaccine is assumed to provide partial protection against incident infection (efficacy ${ɛ}_{v}$) and to reduce infectiousness by a factor φ<1 [6], [30]. We further assume that recovered individuals may lose immunity and revert to the susceptible class at rate ω [8], [29]. Because pre-exposure vaccination is recommended for groups at elevated risk of mpox exposure [56], pre-exposure vaccination is included only for high-risk adults and for children, at rates $\alpha_{a}^{h}$ and $\alpha_{c}$, respectively. Owing to ongoing community contacts between adults (both risk groups) and children, post-exposure vaccination is included for all age/risk groups at rates $\theta_{a}^{h}$, $\theta_{a}^{l}$ and $\theta_{c}$ for high-risk adults, low-risk adults, and children, respectively.

**Fig. 2:**
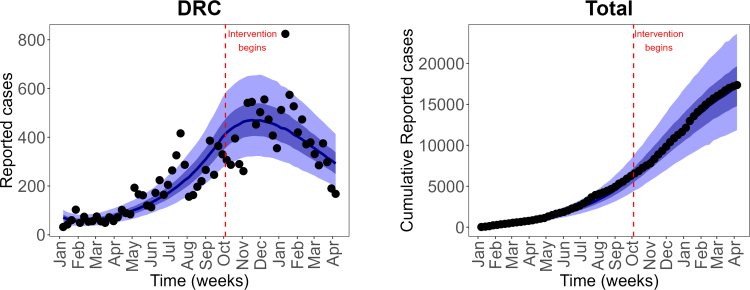
**Reported weekly and predicted weekly mpox cases**. Black dots are the weekly reported mpox cases, and solid blue lines are the median predicted cases. The narrower (darker) bands are the 50% CrI (credible interval), while the wider (lighter) bands are the 95% CrI.

**Fig. 3:**
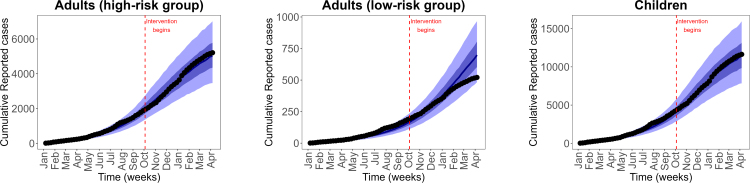
**Reported cumulative and predicted cumulative mpox cases**. Left panel: Adults (high-risk group), middle panel: Adults (low-risk group) and right panel: Children population. Black dots are the weekly reported mpox cases, and solid blue lines are the median predicted cases. The narrower (darker) bands are the 50% CrI (credible interval), while the wider (lighter) bands are the 95% CrI.

## 3 Reproduction number

In this section, we present and give the biological interpretation of the control reproduction number for the model (2.1). The reproduction number is the average number of secondary Mpox infections generated by a single infectious individual in a completely susceptible population [58]. Using the next-generation matrix approach [58], the control reproduction number (where the transfer matrices F and V used in the derivation are given in the Supplementary section) is given by

(3.1) $$R_{0}^{c}=\frac{\left(R_{aa}+R_{cc}\right)+\sqrt{{\left(R_{aa}+R_{cc}\right)}^{2}-4\left(R_{aa}R_{cc}-R_{ac}R_{ca}\right)}}{2},$$

where the control reproduction numbers in the adults-only and children-only populations are defined by $R_{aa}$ and $R_{cc}$ given by

(3.2) $$R_{aa}=\frac{\left(R_{a}^{hh}+R_{a}^{ll}\right)+\sqrt{{\left(R_{a}^{hh}+R_{a}^{ll}\right)}^{2}-4\left(R_{a}^{hh}R_{a}^{ll}-R_{a}^{hl}R_{a}^{lh}\right)}}{2},$$

$$R_{cc}=\beta_{cc}\;\left[\frac{S_{c}^{*}}{N_{c}^{*}\;\left(\sigma_{c}+\theta_{c}\right)}\left(\frac{\sigma_{c}}{\delta_{c}+\gamma_{c}}+\frac{\theta_{c}\;\varphi }{\delta_{c}^{v}+\gamma_{c}^{v}}\right)+\frac{\varphi \left(1-{ɛ}_{v}\right)\;V_{c}^{*}}{N_{c}^{*}\;\left(\delta_{c}^{v}+\gamma_{c}^{v}\right)}\right]$$

where,

$$R_{a}^{hh}=\left(\beta_{a}^{s}+\beta_{a}^{hh}\right)\;\left[\frac{S_{a}^{h*}}{N_{a}^{h*}\;\left(\sigma_{a}^{h}+\theta_{a}^{h}\right)}\left(\frac{\sigma_{a}^{h}}{\delta_{a}^{h}+\gamma_{a}^{h}}+\frac{\theta_{a}^{h}\;\varphi }{\delta_{a}^{hv}+\gamma_{a}^{hv}}\right)+\frac{\varphi \left(1-{ɛ}_{v}\right)\;V_{a}^{h*}}{N_{a}^{h*}\;\left(\delta_{a}^{hv}+\gamma_{a}^{hv}\right)}\right],$$

$$R_{hl}^{a}=\beta_{a}^{hl}\;\left[\frac{\sigma_{a}^{l}}{\left(\sigma_{a}^{l}+\theta_{a}^{l}\right)\;\left(\delta_{a}^{l}+\gamma_{a}^{l}\right)}+\frac{\theta_{a}^{l}\;\varphi }{\left(\sigma_{a}^{l}+\theta_{a}^{l}\right)\;\left(\delta_{a}^{lv}+\gamma_{a}^{lv}\right)}\right],$$

$$R_{a}^{lh}=\beta_{a}^{lh}\;\left[\frac{S_{a}^{h*}}{N_{a}^{h*}\;\left(\sigma_{a}^{h}+\theta_{a}^{h}\right)}\left(\frac{\sigma_{a}^{h}}{\delta_{a}^{h}+\gamma_{a}^{h}}+\frac{\theta_{a}^{h}\;\varphi }{\delta_{a}^{hv}+\gamma_{a}^{hv}}\right)+\frac{\varphi \left(1-{ɛ}_{v}\right)\;V_{a}^{h*}}{N_{a}^{h*}\;\left(\delta_{a}^{hv}+\gamma_{a}^{hv}\right)}\right],$$

$$R_{a}^{ll}=\frac{\beta_{a}^{ll}}{\left(\sigma_{a}^{l}+\theta_{a}^{l}\right)}\left(\frac{\sigma_{a}^{l}}{\delta_{a}^{l}+\gamma_{a}^{l}}+\frac{\theta_{a}^{l}\;\varphi }{\delta_{a}^{lv}+\gamma_{a}^{lv}}\right),$$

$$R_{ca}=\beta_{ca}\;\left[\frac{S_{a}^{h*}}{N_{a}^{h*}\;\left(\sigma_{a}^{h}+\theta_{a}^{h}\right)}\left(\frac{\sigma_{a}^{h}}{\delta_{a}^{h}+\gamma_{a}^{h}}+\frac{\theta_{a}^{h}\;\varphi }{\delta_{a}^{hv}+\gamma_{a}^{hv}}\right)+\frac{\varphi \left(1-{ɛ}_{v}\right)\;V_{a}^{h*}}{N_{a}^{h*}\;\left(\delta_{a}^{hv}+\gamma_{a}^{hv}\right)}\right]+\frac{\beta_{ca}}{\left(\sigma_{a}^{l}+\theta_{a}^{l}\right)}\left(\frac{\sigma_{a}^{l}}{\delta_{a}^{l}+\gamma_{a}^{l}}+\frac{\theta_{a}^{l}\;\varphi }{\delta_{a}^{lv}+\gamma_{a}^{lv}}\right),$$

$$R_{ac}=\beta_{ac}^{h}\left[\frac{S_{c}^{*}}{N_{c}^{*}\;\left(\sigma_{c}+\theta_{c}\right)}\left(\frac{\sigma_{c}}{\delta_{c}+\gamma_{c}}+\frac{\theta_{c}\;\varphi }{\delta_{c}^{v}+\gamma_{c}^{v}}\right)+\frac{\varphi \left(1-{ɛ}_{v}\right)\;V_{c}^{*}}{N_{c}^{*}\;\left(\delta_{c}^{v}+\gamma_{c}^{v}\right)}\right]+\beta_{ac}^{l}\left[\frac{S_{c}^{*}}{N_{c}^{*}\;\left(\sigma_{c}+\theta_{c}\right)}\left(\frac{\sigma_{c}}{\delta_{c}+\gamma_{c}}+\frac{\theta_{c}\;\varphi }{\delta_{c}^{v}+\gamma_{c}^{v}}\right)+\frac{\varphi \left(1-{ɛ}_{v}\right)\;V_{c}^{*}}{N_{c}^{*}\;\left(\delta_{c}^{v}+\gamma_{c}^{v}\right)}\right],$$

where

$$N_{a}^{h*}=S_{a}^{h*}+V_{a}^{h*},\;N_{c}^{*}=S_{c}^{*}+V_{c}^{*},\;V_{a}^{h*}=\alpha_{a}^{h}\;S_{a}^{h*},\;S_{a}^{l*}=N_{a}^{l*},$$

$$V_{c}^{*}=\alpha_{c}\;S_{c}^{*}.$$

The term

$$\left(\beta_{a}^{s}+\beta_{a}^{hh}\right)\frac{S_{a}^{h*}}{N_{a}^{h*}\left(\sigma_{a}^{h}+\theta_{a}^{h}\right)}\frac{\sigma_{a}^{h}}{\delta_{a}^{h}+\gamma_{a}^{h}}$$

represents the average number of secondary mpox infections generated through sexual and non-sexual contacts by a single initially susceptible high-risk adult who acquires mpox, becomes exposed, and progresses to the unvaccinated infectious stage via the route

$$S_{a}^{h}\to E_{a}^{h}\to I_{a}^{h},$$

during their infectious period in the susceptible high-risk subpopulation. Similarly, the term

$$\left(\beta_{a}^{s}+\beta_{a}^{hh}\right)\frac{S_{a}^{h*}}{N_{a}^{h*}\left(\sigma_{a}^{h}+\theta_{a}^{h}\right)}\frac{\theta_{a}^{h}\varphi }{\delta_{a}^{hv}+\gamma_{a}^{hv}}$$

represents the average number of secondary mpox infections resulting from sexual and non-sexual contacts by one initially susceptible high-risk adult who acquires mpox, becomes exposed, and progresses to the vaccinated infectious stage via the route

$$S_{a}^{h}\to E_{a}^{h}\to I_{a}^{hv},$$

during their infectious period in the susceptible high-risk subpopulation. The third term in $R_{hh}^{c}$,

$$\left(\beta_{a}^{s}+\beta_{a}^{hh}\right)\frac{\varphi \left(1-{ɛ}_{v}\right)V_{a}^{h*}}{N_{a}^{h*}\left(\delta_{a}^{hv}+\gamma_{a}^{hv}\right)},$$

represents the average number of secondary mpox infections arising from sexual and non-sexual contacts by one initially vaccinated high-risk adult who acquires mpox, becomes exposed, and progresses to the vaccinated infectious stage via the route

$$V_{a}^{h}\to E_{a}^{hv}\to I_{a}^{hv},$$

during their infectious period in the susceptible high-risk subpopulation. Other terms in the components of the control reproduction number, $R_{0}^{c}$, can be interpreted in a similar manner.

Epidemiologically, the threshold quantity $R_{aa}$ represents the average number of secondary mpox infections generated by a single infectious adult in a wholly susceptible adult population, while $R_{cc}$ denotes the corresponding measure for an infectious child in a entirely susceptible children population. The cross-age transmission terms capture intergroup infection potential, where: $R_{ac}$ is the expected number of secondary adult cases produced by one infectious child in a totally susceptible adult population, and $R_{ca}$ is the expected number of secondary mpox cases generated by one infectious adult in a fully susceptible children population. Within the adult population, further stratification into high-risk and low-risk groups allows for a more refined characterization of transmission pathways. In particular, $R_{a}^{hh}$ denotes the mean number of secondary mpox infections arising from a single infectious high-risk adult within a completely susceptible high-risk adult population, whereas $R_{a}^{lh}$ quantifies the infections generated by an infectious high-risk adult in a wholly susceptible low-risk adult population. Conversely, $R_{a}^{hl}$ represents the average number of secondary infections generated by a single infectious low-risk adult in a completely susceptible high-risk adult population, and $R_{a}^{ll}$ reflects the secondary infections generated by an infectious low-risk adult in a fully susceptible low-risk adult population.

**Fig. 4:**
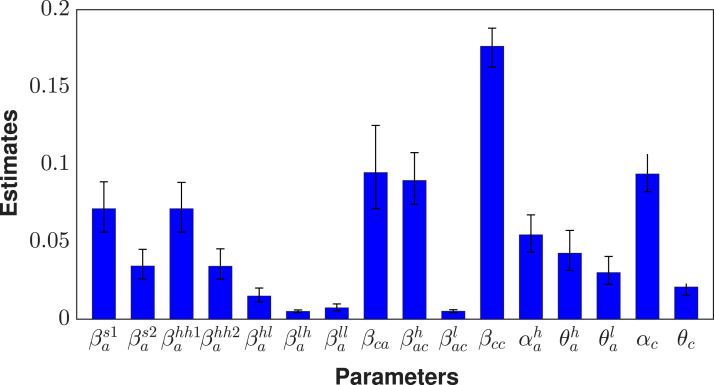
**Estimated parameters**. Estimated parameters with 95% CrI (credible interval). Here, $\beta_{a}^{s1}=\beta_{a}^{s}\left(t\right)$ and $\beta_{a}^{hh1}=\beta_{a}^{hh}\left(t\right)$ when $t\leq t_{a}$ while $\beta_{a}^{s2}=\beta_{a}^{s}\left(t\right)$ and $\beta_{a}^{hh2}=\beta_{a}^{hh}\left(t\right)$ when $t>t_{a}$.

**Fig. 5:**
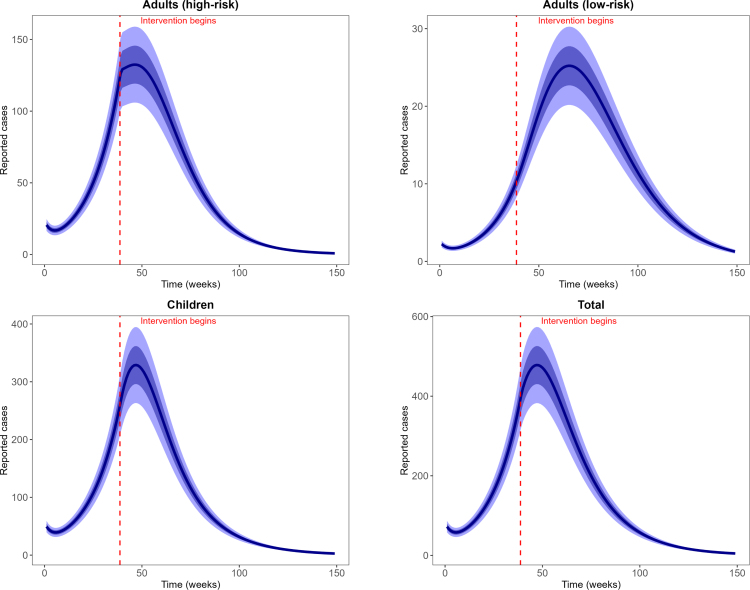
**Predicted weekly incidence**. Predicted weekly incidence for adults (high-risk group), adults (low-risk group), children and total population. Solid blue lines are the median predicted weekly incidence. The narrower (darker) bands are the 50% CrI (credible interval), while the wider (lighter) bands are the 95% CrI.

**Fig. 6:**
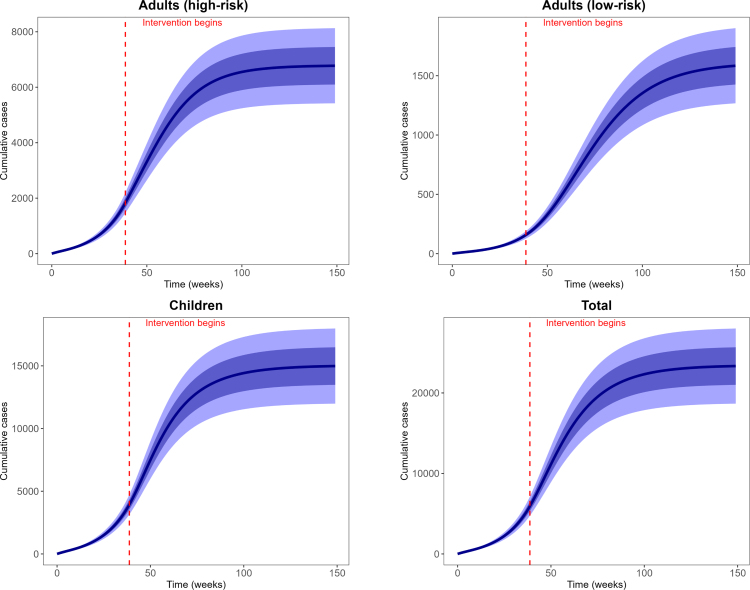
**Predicted cumulative cases**. Predicted cumulative cases for adults (high-risk group), adults (low-risk group), children and total population. Solid blue lines are the median predicted cumulative cases. The narrower (darker) bands are the 50% CrI (credible interval), while the wider (lighter) bands are the 95% CrI.

**Fig. 7:**
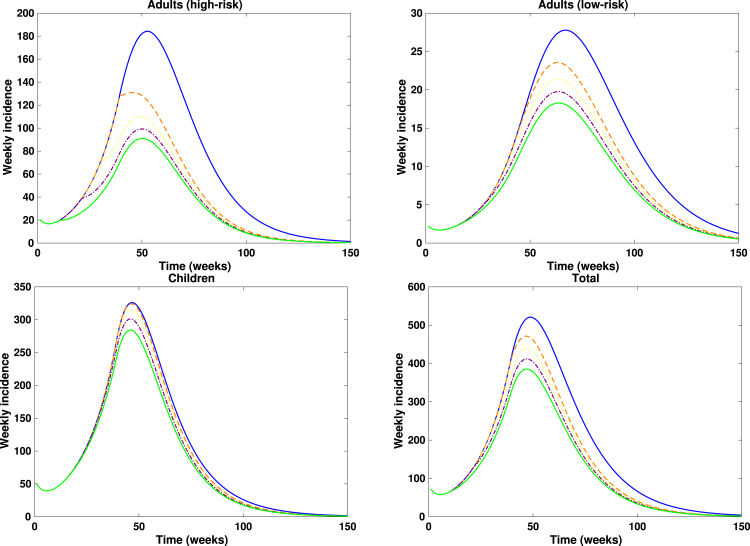
**Impact of timing of reduction in contacts among adults (high-risk group)**. Weekly incidence of mpox when the timing of reduction in contacts is varied while fixing the timing of vaccination intervention at $t_{a}$.

**Fig. 8:**
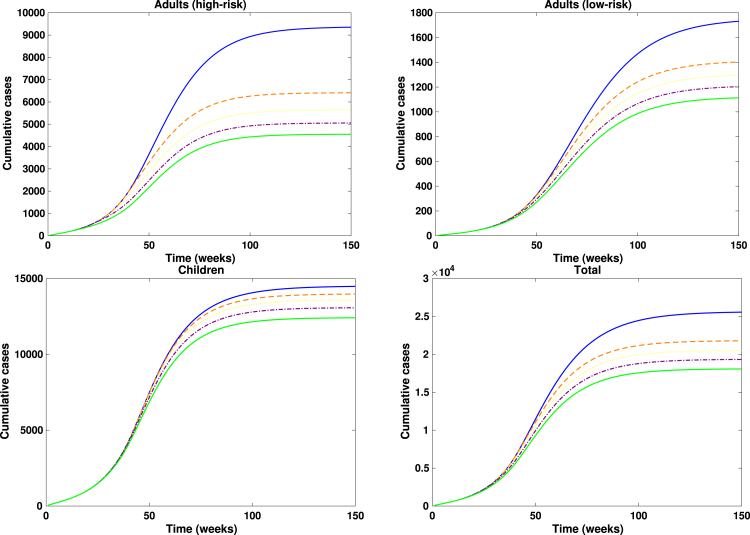
**Impact of timing of reduction in contacts among adults (high-risk group)**. Cumulative cases of mpox when the timing of reduction in contacts is varied while fixing the timing of vaccination intervention at $t_{a}$.

**Fig. 9:**
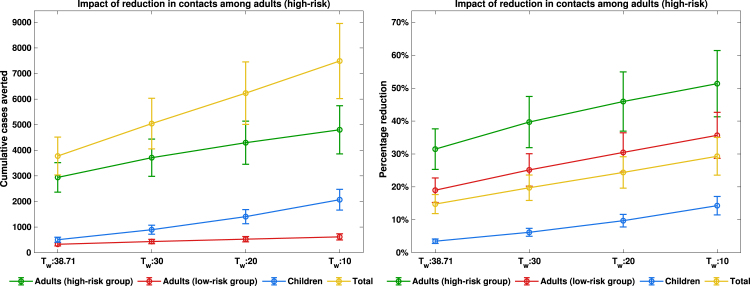
**Impact of reduction in contacts among adults (high-risk)**. Peak magnitudes and final cumulative cases.

**Fig. 10:**
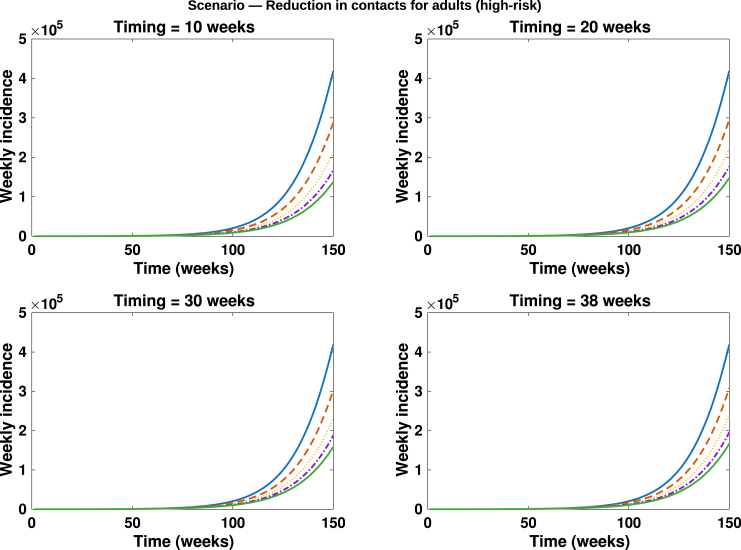
**Reduction in contacts among adults (high-risk group)**. Simulated total weekly incidence to assess the impact of timing and magnitude of reduction in contacts among adults (high-risk group). Each curve represents the effect of contact reduction at different intensity levels: blue solid lines: 0%, orange dashed lines: 20%, yellow dotted lines: 40%, purple dash dotted lines: 60% and green solid lines: 80%. A higher percentage corresponds to stronger behavioural change.

**Fig. 11:**
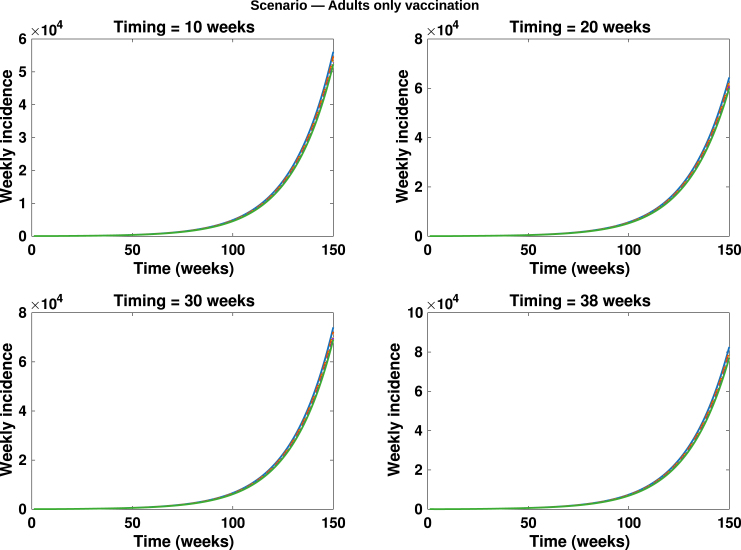
**Adults only vaccination**. Simulated total weekly incidence to assess the impact of timing and magnitude of adults only vaccination. Each curve represents the effect of vaccination increase at different intensity levels: blue solid lines: 0%, orange dashed lines: 20%, yellow dotted lines: 40%, purple dash dotted lines: 60% and green solid lines: 80%. A higher percentage corresponds to greater vaccination uptake.

**Fig. 12:**
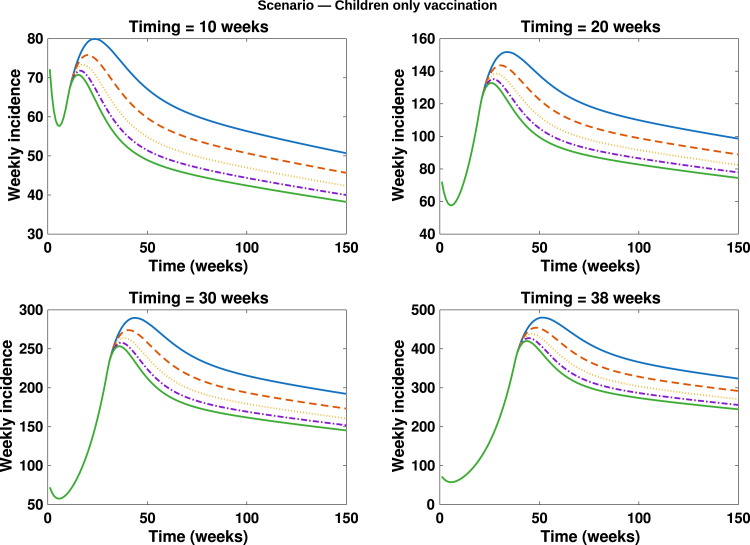
**Children only vaccination**. Simulated total weekly incidence to assess the impact of children only vaccination timing and magnitude. Each curve represents the effect of vaccination increase at different intensity levels: blue solid lines: 0%, orange dashed lines: 20%, yellow dotted lines: 40%, purple dash dotted lines: 60% and green solid lines: 80%. A higher percentage corresponds to greater vaccination uptake.

**Fig. 13:**
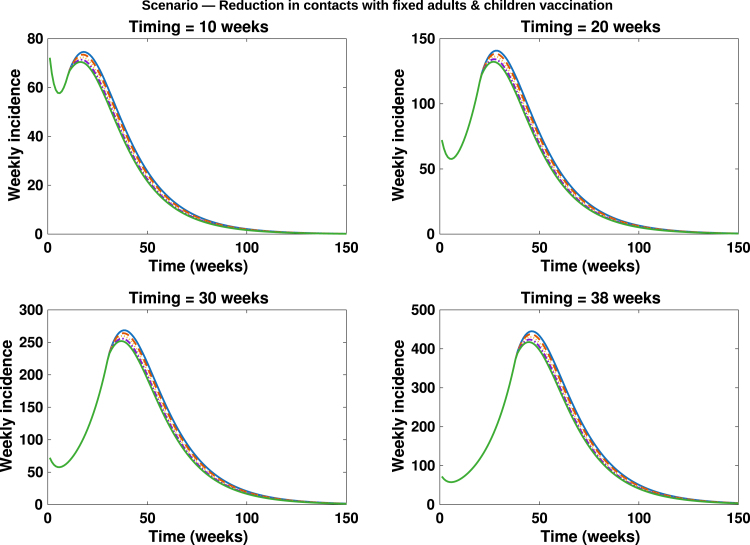
**Contacts reduction with fixed adults and children vaccination**. Simulated total weekly incidence to assess the effect of contacts reduction among adults (high-risk group) with fixed adults and children vaccination. Each curve represents effect of contact reduction at different intensity levels: blue solid lines: 0%, orange dashed lines: 20%, yellow dotted lines: 40%, purple dash dotted lines: 60% and green solid lines: 80%. A higher percentage corresponds to stronger behavioural change.

**Fig. 14:**
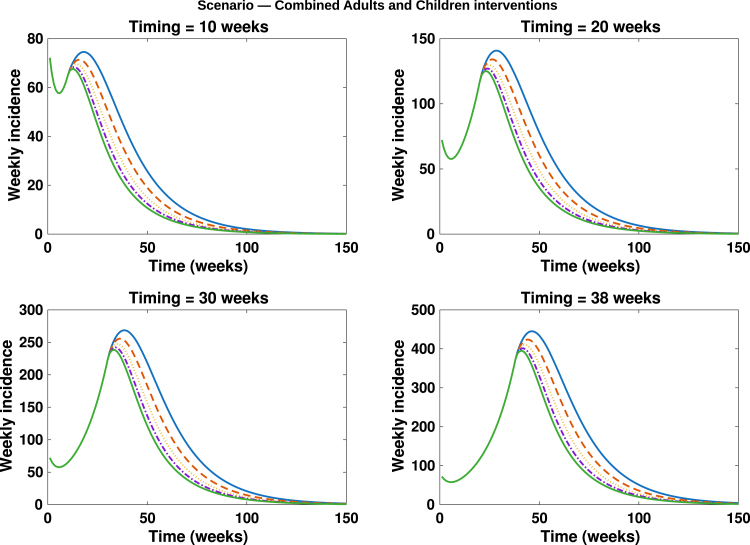
**Combined adults and children intervention**. Simulated total weekly incidence to assess the combined effect of adult and children intervention timing and magnitude. Each curve represents a combined effect of contact reduction and vaccination increase at different intensity levels: blue solid lines: 0%, orange dashed lines: 20%, yellow dotted lines: 40%, purple dash dotted lines: 60% and green solid lines: 80%. A higher percentage corresponds to stronger behavioural change and vaccination uptake.

## 4 Results

### 4.1 Model fitting

In this subsection, we present key results from the calibration of our model to DRC mpox data. We calibrated our vaccination model (2.1) to the weekly reported mpox cases using a Bayesian inference framework and the RStan package in R version 3.6.3 [59].

In the model, $\alpha_{a}^{h}E_{a}^{h}\left(t\right)+\alpha_{a}^{hv}E_{a}^{hv}\left(t\right)$, $\alpha_{a}^{l}E_{a}^{l}\left(t\right)+\alpha_{a}^{lv}E_{a}^{lv}\left(t\right)$ and $\alpha_{c}E_{c}\left(t\right)+\alpha_{c}^{v}E_{c}^{v}\left(t\right)$ are the weekly mpox incidence for the adults (high-risk group), adults (low-risk group) and children, respectively, which we calibrated to the combined weekly reported mpox cases in the DRC using the likelihood functions:

(4.1) HighRisk_cases(t)∼NegBin(IncidenceAdultsHighRisk(t)×ϕ,ϕ)AdultsLowRisk_cases(t)∼NegBin(IncidenceLowRisk(t)×ϕ,ϕ)Children_cases(t)∼NegBin(IncidenceChildren(t)×ϕ,ϕ)

where AdultsHighRisk_cases(t), AdultsLowRisk_cases(t) and Children_cases(t) are the weekly reported mpox cases for adults (high-risk group), adults (low-risk group) and children, respectively. NegBin(⋅) is the Negative binomial distribution with shape parameters IncidenceAdultsHighRisk(t)×ϕ, IncidenceAdultsLowRisk(t)×ϕ and IncidenceChildren(t)×ϕ for the adults (high-risk group), adults (low-risk group) and children, respectively and ϕ is the overdispersion parameter.

For our model calibration, we used weekly reported mpox cases from the Democratic Republic of the Congo (DRC), covering the period from January 7, 2024 to April 6, 2025 [60]. Because these data are not stratified by age, we allocated 67% of reported cases to children and 30% and 3% to high-risk and low-risk adults, respectively, following the age distribution of mpox cases reported by the World Health Organization [25], [61]. We also incorporated demographic information indicating that children under 15 years constitute approximately 46% of the DRC population, while high-risk and low-risk adults represent 19% and 35% of the population, respectively [11]. Given the total DRC population N(0)=105,044,646, the corresponding initial sub-populations were calculated as follows: high-risk adults $N_{a}^{h}\left(0\right)=19,853,438$, low-risk adults $N_{a}^{l}\left(0\right)=36,870,670$, and children $N_{c}\left(0\right)=48,320,537$. Using these initial conditions, we calibrated the model, and the resulting parameter estimates are presented in Figs. 2, 3, and 4. The estimated median sexual transmission rate before behavioural change was $\beta_{a}^{s}=0.07149$ (95% CrI: 0.056271–0.088791), decreasing to $\beta_{a}^{s}=0.034366$ (95% CrI: 0.026063–0.045170) after behavioural change. Similarly, the median community-based transmission rate within the high-risk adult group was $\beta_{a}^{hh}=0.071422$ (95% CrI: 0.056374–0.088447) before behavioural change and $\beta_{a}^{hh}=0.034255$ (95% CrI: 0.025807–0.045478) after behavioural change. Other estimated transmission rates involving adults were $\beta_{a}^{hl}=0.01499$ (95% CrI: 0.011156–0.020128), $\beta_{a}^{lh}=0.005094$ (95% CrI: 0.004243–0.006010), and $\beta_{a}^{ll}=0.007378$ (95% CrI: 0.005529–0.009850). For child–adult interactions, the estimated median transmission rate from infectious adults to susceptible children was $\beta_{ca}=0.094789$ (95% CrI: 0.071432–0.125235), while transmission among children was $\beta_{cc}=0.17638$ (95% CrI: 0.162978–0.188059). Transmission from infectious children to adults in the high- and low-risk groups was estimated as $\beta_{ac}^{h}=0.089658$ (95% CrI: 0.074297–0.107653) and $\beta_{ac}^{l}=0.005178$ (95% CrI: 0.004294–0.006150), respectively. The estimated median initial numbers of exposed individuals were $E_{a}^{h}\left(0\right)=184$ (95% CrI: 166–204), $E_{a}^{l}\left(0\right)=19$ (95% CrI: 16–23), and $E_{c}\left(0\right)=442$ (95% CrI: 411–476). The predicted weekly incidence and cumulative cases, based on the estimated parameters, are presented in Fig. 5 and Fig. 6, respectively.

Additionally, the sensitivity analysis of the control reproduction number and the epidemic peak size are presented in Fig. S.1 in the Supplementary.

### 4.2 Scenario analysis

In this section, we perform a scenario analysis of our model (2.1) to optimize vaccination strategies for mpox outbreak containment in the Democratic Republic of the Congo (DRC).

The scenario analysis assessing the impact of contact reduction among adults in the high-risk group is presented in Fig. 7, Fig. 8. In these figures, the solid blue lines denote interventions initiated at the baseline time (t=38.71 weeks), the orange dashed lines correspond to interventions implemented at t=30 weeks, the purple dash-dotted lines represent reductions initiated at t=20 weeks, while the solid green lines illustrate reductions beginning at t=10 weeks. Overall, a pronounced decline in both the epidemic peak and the cumulative number of mpox cases is observed across adults in the high-risk and low-risk subpopulations, as well as in the general population. In contrast, only marginal reductions are evident among children, which is expected since the behavioural modification was confined to adult groups. Notably, implementing a 50% reduction in contacts approximately 20 weeks earlier among high-risk adults led to an additional 20% decrease in cumulative mpox cases compared with scenarios where contact reduction coincided with the initiation of vaccination in the DRC (see Fig. 9). This result underscores the critical importance of timely behavioural interventions in curbing mpox transmission, consistent with previous findings that early behavioural change can substantially attenuate epidemic trajectories in high-transmission networks [62], [63], [64].

**Fig. S.2:**
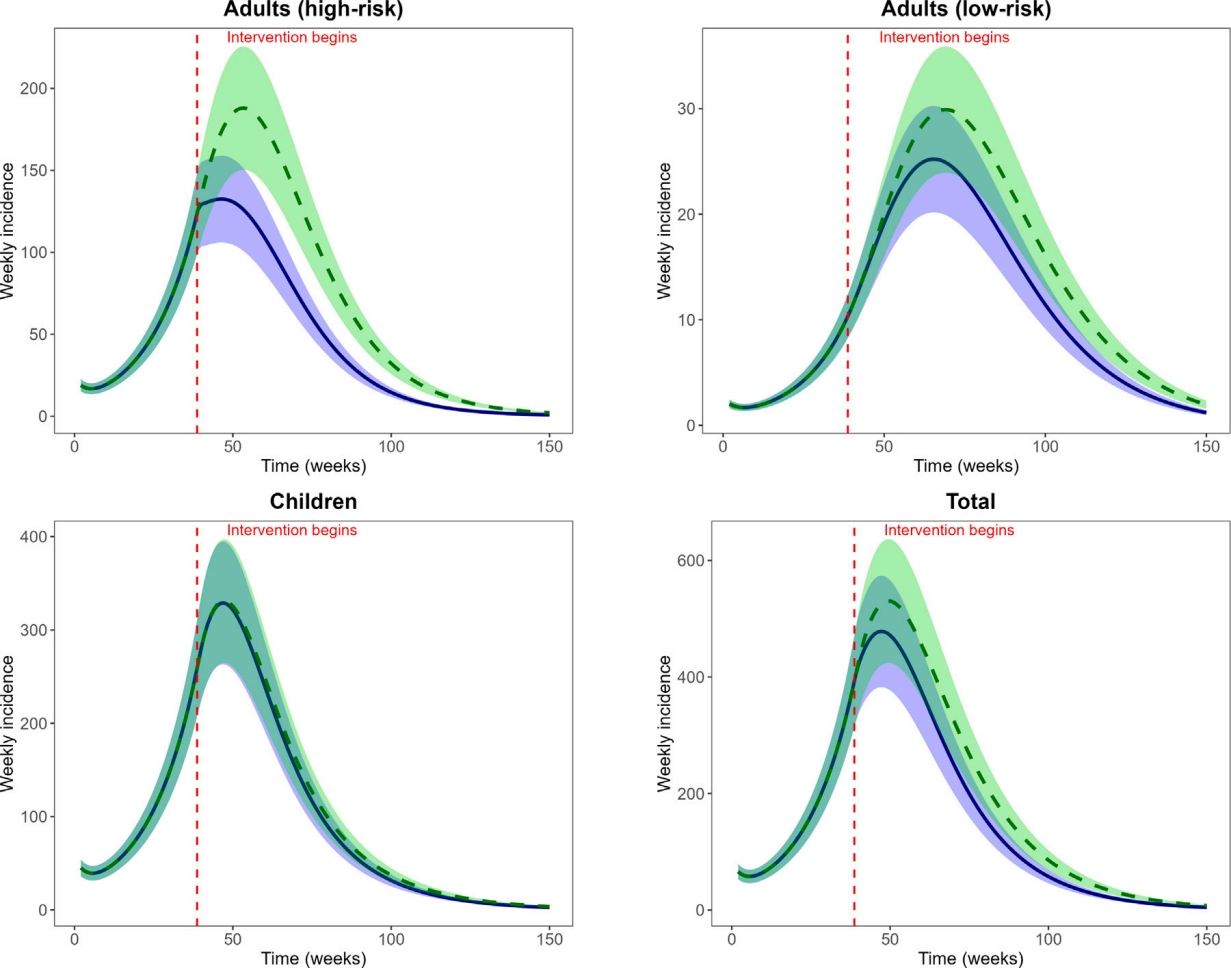
**Impact of reduction in contacts among adults (high-risk group)**. Weekly incidence of mpox to assess the impact of contacts reduction among adults (high-risk group). The blue solid line represents the scenario with adults high-risk group contacts reduction, while the green dashed line represents the scenario without contacts reduction. Shaded areas indicate 95% credible intervals around the median estimates. The vertical red dashed line marks the start of the intervention.

**Fig. S.3:**
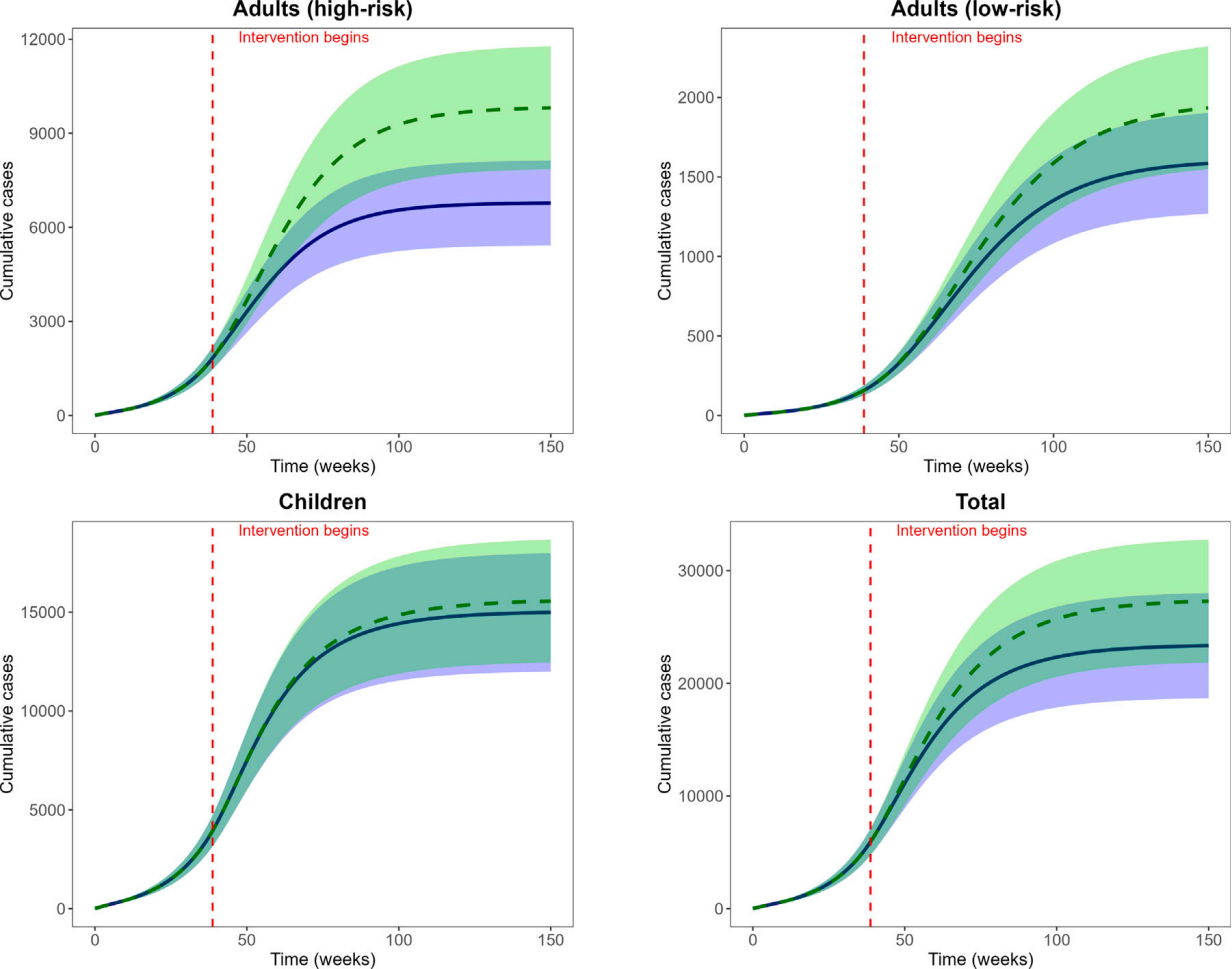
**Impact of reduction in contacts among adults (high-risk group)**. Cumulative cases of mpox to assess the impact of contacts reduction among adults (high-risk group). The blue solid line represents the scenario with adults high-risk group contacts reduction, while the green dashed line represents the scenario without contacts reduction. Shaded areas indicate 95% credible intervals around the median estimates. The vertical red dashed line marks the start of the intervention.

In Fig. 10, Fig. 11, Fig. 12, Fig. 13, Fig. 14, we evaluate the effects of five distinct intervention scenarios designed to assess the impact of vaccination and behavioural modification across different age groups in the Democratic Republic of the Congo (DRC). In Fig. 10, we investigate the effects of contact reduction among adults in the high-risk group. The results show that the total cumulative number of mpox cases decreases substantially with increasing intensity of behavioural change and earlier implementation of interventions. This finding underscores the importance of reducing risky contacts among adults in the high-risk group, who constitute the primary transmitters in most endemic settings [63], [65]. Similar patterns have been reported in studies showing that behavioural adaptations – such as reductions in physical and sexual contacts – can significantly mitigate mpox transmission [62], [66]. Simulations illustrating the effects of this intervention on the peak magnitudes and cumulative cases (including the 50% credible intervals) for the different subgroups – high-risk adults, low-risk adults, children, and the total population – are provided in the Supplementary Fig. S.2, Fig. S.3. In Fig. 11, we assess the impact of an adults-only vaccination strategy. The results indicate only a marginal reduction in cumulative cases, even at higher coverage levels. This suggests that vaccination among adults in the high-risk group yields a relatively lower impact compared to behavioural change within the same group. In Fig. 12, we examine the effects of a childhood-focused vaccination strategy. This approach demonstrates markedly stronger control outcomes compared with the adults-only vaccination strategy, reflecting the greater contribution of children to overall mpox transmission dynamics in the DRC. In Fig. 13, we evaluate the combined impact of contact reduction among high-risk adults with fixed vaccination coverage in both adults and children. This integrated intervention produces a synergistic reduction in mpox cases. The combined effects of behavioural modification (among high-risk adults) and vaccination coverage greatly enhance disease control outcomes, demonstrating that even moderate behavioural changes, when coupled with vaccination, can substantially reduce mpox transmission. These findings are consistent with previous modelling studies in the literature [67].

In Fig. 14, we investigate the combined effect of adult and children intervention measures. It is observed that this strategy recorded the greatest overall reduction in cumulative mpox cases, compared to other intervention measures. Thus, emphasizing the importance of integrating both behavioural adaptation and vaccination across all age groups. For instance, with 80% intervention intensity implemented at the 10th week, we observed a about 32% reduction in cumulative cases. This intervention strategy not only speed up epidemic decline but also enhances community-level immunity and resilience against potential mpox resurgence. These findings reinforce the consensus that multi-layered public health strategies are more effective in mitigating emerging infectious diseases [62], [68]. The associated cumulative mpox cases under the five distinct intervention scenarios are presented in the Supplementary Table S.3, Table S.4, Table S.5, Table S.6, Table S.7. Overall, our results reveal that early and sustained behavioural interventions, when combined with age-targeted vaccination, can significantly lower mpox transmission in the DRC.

## 5 Discussion and conclusion

The high profiles of cases among children (especially those below the age of 15) and adults with occupational risks (particularly sex workers and miners) during the recent mpox clade I outbreak in the DRC have revealed distinct transmission dynamics and risk factors that highlight the vulnerability of these populations [23], [26]. This underscores the need for targeted public health interventions and rapid response mechanisms to curb the spread of mpox within these vulnerable populations. In this paper, we develop a compartmental age model to understand the dynamics of mpox in the DRC. The model categorizes the population into adults (high- and low-risk groups) and children. Additionally, our model incorporates pre-exposure vaccination for adults in the high-risk group and children and post-exposure vaccination for individuals in all age-groups.

The model is calibrated to the weekly reported mpox cases for the DRC from January 2024 to April 2025, from which we estimated key parameters of the model such as the sexual transmission rate among adults in the high-risk group and transmission rates among the adults and children populations. The observed higher transmission rates among adults in the high-risk group and among children population is not surprising. Firstly, children often rely heavily on adults for care, leading to close, frequent contact, especially if an adult in the household is infected. In communities where mpox is endemic, there tends to be higher transmission among children and high-risk adults [69]. In high-risk adult groups, exposure to the virus through occupational or environmental factors means they are more likely to bring the virus into households. Children then become vulnerable due to their close physical interactions with adults and other children.

From the scenario analyses, our findings reveal that the children-focused vaccination strategy yields greater epidemiological benefits compared to the adults-only vaccination strategy. This result aligns with existing reports indicating that approximately 67% of confirmed mpox cases in the DRC have occurred among children [25]. Consequently, children constitute a substantial proportion of the high-risk susceptible population and serve as key drivers in the transmission chain. Vaccinating children therefore markedly reduces the susceptible pool within this age group, effectively disrupting mpox transmission pathways. By prioritizing vaccination among children, the intervention achieves a significant reduction in both the epidemic peak and the cumulative number of cases. In contrast, a vaccination strategy focusing solely on adults results in a comparatively smaller impact. Thus, a children-focused vaccination approach not only protects those at the highest risk of mpox infection but also strengthens population-level immunity, leading to a greater overall reduction in mpox incidence. These results are consistent with previous studies emphasizing the importance of prioritizing childhood vaccination to effectively control mpox in endemic regions [26], [53], [54].

Overall, the results from our study indicate that behavioural change among adults in the high-risk group could play a significant role in curtailing mpox transmission in endemic regions. Furthermore, the simulation results show that contact reductions among high-risk adults lead to a faster and greater decline in both the epidemic peak and cumulative mpox cases compared with vaccination alone within the same age group. These findings highlight that, when sustained over a long period, behavioural modification can enhance long-term mpox control. Insights from this study are particularly relevant in the context of the DRC, where vaccination coverage remains very low [70]. Collectively, our findings provide critical evidence to guide the strategic prioritization of vaccination and behavioural interventions in resource-limited settings.

### Limitations

This study has several limitations. First, the model assumes homogeneous mixing within age groups and does not incorporate dynamic changes in population demographics or transitions between risk groups. We did not emphasize these processes because they fall outside the primary scope of this analysis, although their inclusion could refine short- and long-term projections. Second, we did not explicitly represent heterogeneity in non-sexual contact rates and patterns across the population. Non-sexual transmission was modelled collectively rather than distinguishing household- versus community-based transmission pathways, which may overlook important differences in exposure risk and clustering. These simplifications were adopted to preserve model tractability and to focus on the comparative effects of vaccination and behaviour-driven contact reduction. Nevertheless, they may constrain the precision and context-specific applicability of our quantitative predictions. Future work that relaxes these assumptions – for example, by incorporating age-structured contact matrices, explicit household structure, demographic dynamics, and behavioural transitions between risk classes – could produce more locally tailored and actionable guidance for public-health authorities managing mpox in endemic settings.

## CRediT authorship contribution statement

**Andrew Omame:** Writing – review & editing, Writing – original draft, Visualization, Validation, Software, Resources, Methodology, Investigation, Formal analysis, Data curation, Conceptualization. **Nicola Luigi Bragazzi:** Writing – review & editing, Writing – original draft, Validation, Supervision, Resources, Project administration, Investigation, Funding acquisition, Data curation, Conceptualization. **Ali Asgary:** Validation, Supervision, Project administration, Investigation, Funding acquisition, Data curation, Conceptualization. **Chigozie Louisa J. Ugwu:** Writing – review & editing, Writing – original draft, Methodology, Investigation, Funding acquisition, Formal analysis, Data curation, Conceptualization. **Jude Dzevela Kong:** Visualization, Validation, Supervision, Resources, Project administration, Investigation, Funding acquisition, Formal analysis, Conceptualization. **Jianhong Wu:** Visualization, Validation, Supervision, Resources, Project administration, Investigation, Funding acquisition, Formal analysis, Data curation, Conceptualization. **Woldegebriel Assefa Woldegerima:** Writing – review & editing, Writing – original draft, Validation, Supervision, Software, Resources, Project administration, Investigation, Funding acquisition, Formal analysis, Conceptualization.

## Funding statement

This research is funded by the 10.13039/501100000024Canadian Institute for Health Research (CIHR) under the Mpox and other zoonotic threats Team Grant (FRN. 187246). W.A.W acknowledges financial support from the NSERC Discovery Grant (Appl No.: RGPIN-2023-05100).

## Declaration of competing interest

The authors declare that there is no competing interest.

## Figures and Tables

**Fig. S.1:**
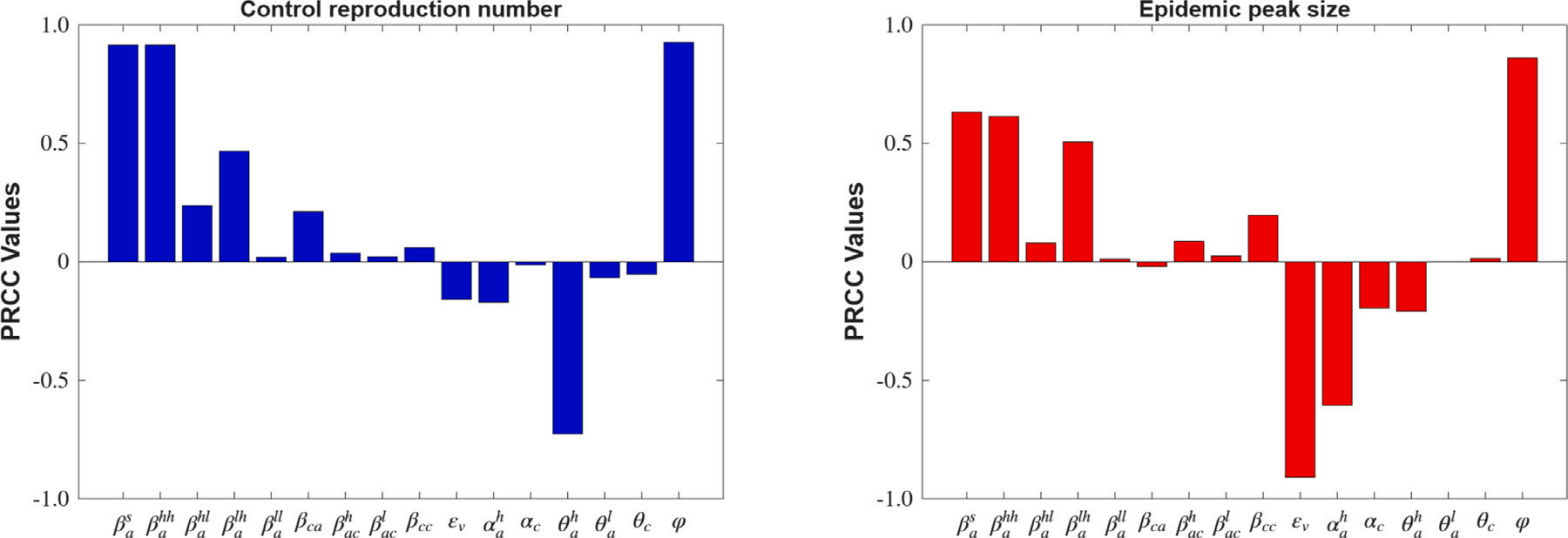
**Sensitivity analysis**. **Left panel**: Sensitivity analysis of the control reproduction number. **Right panel**: Sensitivity analysis of the epidemic peak size of total infected individuals.

**Table S.1:** Parameter distributions used in sensitivity analysis.

Parameter	Lower bound	Upper bound	Distribution
$\beta_{a}^{s}$	0.1	0.5	Uniform
$\beta_{a}^{hh}$	0.1	0.5	Uniform
$\beta_{a}^{hl}$	0.1	0.3	Uniform
$\beta_{a}^{lh}$	0.001	0.003	Uniform
$\beta_{a}^{ll}$	0.001	0.003	Uniform
$\beta_{ac}^{h}$	0.01	0.03	Uniform
$\beta_{ac}^{l}$	0.01	0.03	Uniform
$\beta_{ca}$	0.01	0.1	Uniform
$\beta_{cc}$	0.1	0.3	Uniform
${ɛ}_{v}$	0	1.0	Triangular
φ	0	1.0	Triangular
$\theta_{a}^{h}$	0.001	0.005	Uniform
$\theta_{a}^{l}$	0.001	0.005	Uniform
$\theta_{c}$	0.001	0.005	Uniform
ξ	$\frac{1}{350}$	$\frac{1}{150}$	Uniform
$\alpha_{a}^{h}$	0.01	0.05	Uniform
$\alpha_{c}$	0.01	0.05	Uniform

**Fig. S.4:**
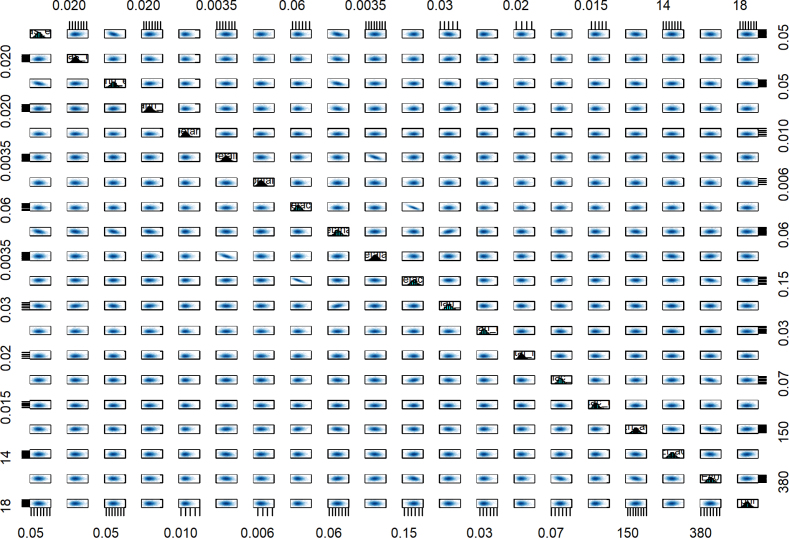
Posterior parameter pairs plots with 5000 iterations and 4 chains for the model.

**Fig. S.5:**
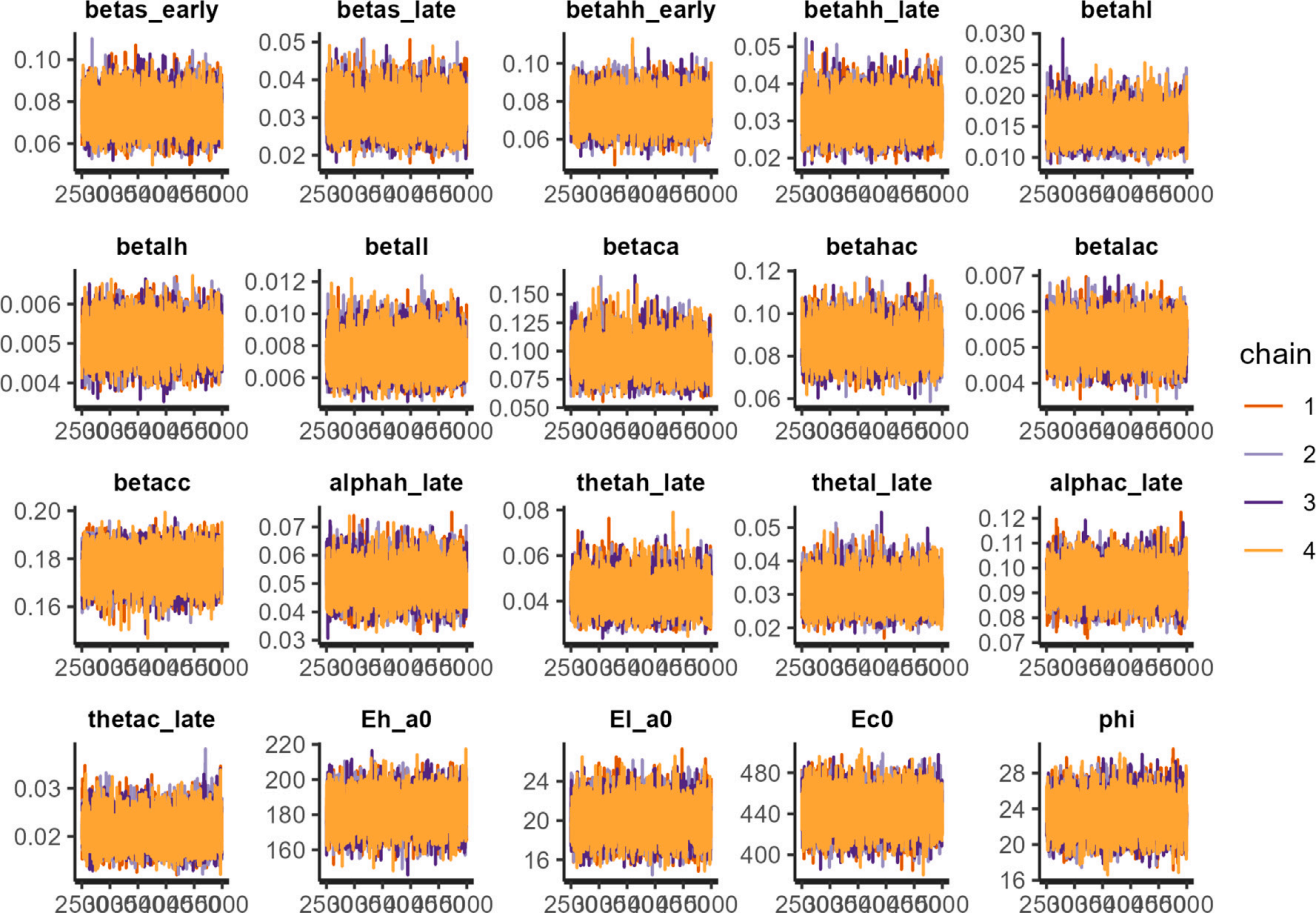
Posterior parameter trace plots with 5000 iterations and 4 chains for the model.

**Fig. S.6:**
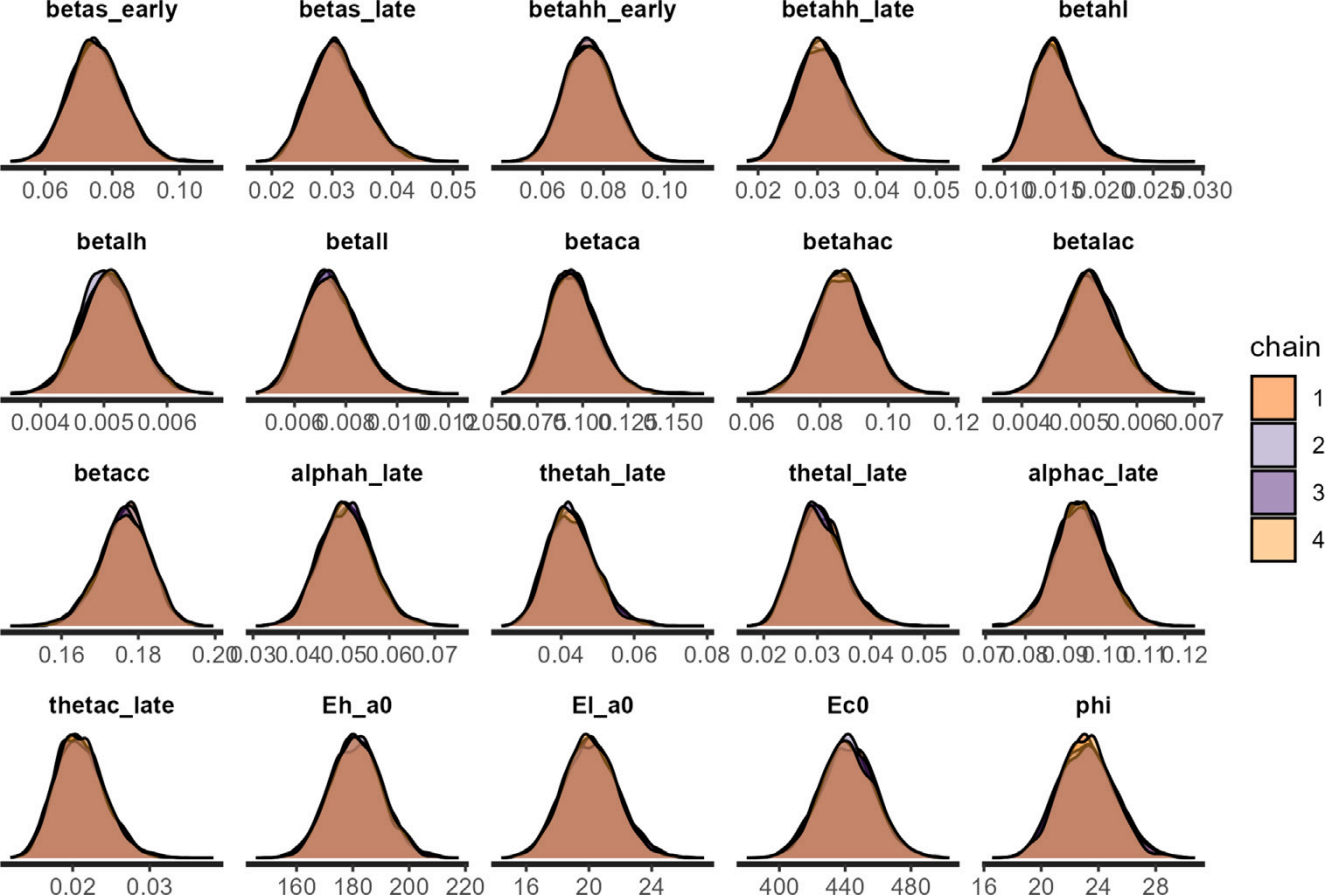
Posterior parameter density plots with 5000 iterations and 4 chains for the model.

**Table S.2:** Posterior distributions from the model fitting. Here, $\beta_{a}^{s1}\left(t\right)=\beta_{a}^{s}\left(t\right)$ and $\beta_{a}^{hh1}\left(t\right)=\beta_{a}^{hh}\left(t\right)$ when $t\leq t_{a}$ while $\beta_{a}^{s2}\left(t\right)=\beta_{a}^{s}\left(t\right)$ and $\beta_{a}^{hh2}\left(t\right)=\beta_{a}^{hh}\left(t\right)$ when $t>t_{a}$, ϕ is the dispersion parameter.

	Mean	SEMean	SD	2.50%	25%	50%	75%	97.50%	$n_{eff}$	Rhat
$\beta_{a}^{s1}$	0.072	0.000089	0.00838	0.0563	0.0659	0.07141	0.0771	0.0887	8805.0	1.00
$\beta_{a}^{s2}$	0.0347	0.000046	0.00500	0.0261	0.0311	0.0344	0.0378	0.0451	11 635.3	1.00
$\beta_{a}^{hh1}$	0.0717	0.000089	0.00826	0.0564	0.0659	0.0714	0.0771	0.0884	8469.0	1.00
$\beta_{a}^{hh2}$	0.0346	0.000047	0.0049	0.0258	0.0312	0.0342	0.0377	0.0454	10 940.3	1.00
$\beta_{a}^{hl}$	0.0151	0.000020	0.00233	0.0112	0.0135	0.0149	0.0167	0.0201	13 232.0	1.00
$\beta_{a}^{lh}$	0.00511	0.0000049	0.000448	0.004243	0.004805	0.005094	0.005402	0.00601	8237.2	1.00
$\beta_{a}^{ll}$	0.00745	0.0000099	0.00109	0.00552	0.00669	0.00737	0.00812	0.00985	12 129.8	1.00
$\beta_{ca}$	0.0956	0.00016	0.0138	0.0714	0.085855	0.0947	0.104	0.125	6674.6	1.00
$\beta_{ac}^{h}$	0.0901	0.00010	0.00851	0.0742	0.0842	0.0896	0.0956	0.1076	7173.5	1.00
$\beta_{ac}^{l}$	0.00519	0.0000053	0.00048	0.00429	0.00487	0.00517	0.00550	0.00615	7817.8	1.00
$\beta_{cc}$	0.17613	0.000081	0.00640	0.162978	0.1719	0.1763	0.1805	0.1880	6191.4	1.00
$\alpha_{a}^{h}$	0.0547	0.000063	0.00617	0.0433	0.0505	0.0545	0.0587	0.06742	9339.8	1.00
$\theta_{a}^{h}$	0.0431	0.000057	0.00658	0.0315	0.0385	0.0426	0.0471	0.0573	13 177.3	1.00
$\theta_{a}^{l}$	0.03047	0.000041	0.00457	0.0225	0.0272	0.0301	0.0332	0.0405	12 598.0	1.00
$\alpha_{c}$	0.0940	0.000061	0.00625	0.0824	0.0898	0.0938	0.0981	0.1067	10 383.3	1.00
$\theta_{c}$	0.0211	0.000028	0.00319	0.0155	0.0188	0.0208	0.0230	0.0280	13 079.1	1.00
$E_{a}^{h}\left(0\right)$	184.867	0.0961	9.6223	166.52	178.40	184.69	191.15	204.55	10 008.5	1.00
$E_{a}^{l}\left(0\right)$	20.018	0.0170	1.764	16.74	18.79	19.98	21.185	23.678	10 691.7	1.00
$E_{c}\left(0\right)$	443.10	0.1839	16.62	411.49	431.64	442.83	454.05	476.78	8169.3	1.00
ϕ	23.4321	0.0173	1.948	19.776	22.084	23.368	24.710	27.43	12 557.4	1.00

**Table S.3:** Total cumulative mpox cases computed from the scenario in Fig. 10 (Contacts reduction among adults (high-risk group)). Here, $t_{w}$ represents the intervention week.

Intensity of intervention	$t_{w}$:10	$t_{w}$:20	$t_{w}$:30	$t_{w}$:37.81
0%	7 483 282	7 483 282	7 483 282	7 483 282
20%	5 217 545	5 353 636	5 501 436	5 604 408
40%	3 914 581	4 093 619	4 298 557	4 441 221
60%	3 127 534	3 318 003	3 539 556	3 695 728
80%	2 623 388	2 813 417	3 037 168	3 196 411

**Table S.4:** Total cumulative mpox cases computed from the scenario in Fig. 11 (Adults only vaccination). Here, $t_{w}$ represents the intervention week.

Intensity of intervention	$t_{w}$:10	$t_{w}$:20	$t_{w}$:30	$t_{w}$:37.81
0%	1 188 037	1 369 124	1 581 513	1 771 286
20%	1 157 479	1 333 926	1 540 962	1 726 013
40%	1 135 544	1 308 647	1 511 820	1 693 449
60%	1 118 936	1 289 510	1 489 765	1 668 810
80%	1 105 858	1 274 442	1 472 411	1 649 435

**Table S.5:** Total cumulative mpox cases computed from the scenario in Fig. 12 (Children only vaccination). Here, $t_{w}$ represents the intervention week.

Intensity of intervention	$t_{w}$:10	$t_{w}$:20	$t_{w}$:30	$t_{w}$:37.81
0%	9291	17 037	30 947	49 014
20%	8488	15 608	28 414	45 069
40%	7945	14 639	26 692	42 383
60%	7553	13 939	25 446	40 437
80%	7257	13 409	24 503	38 962

**Table S.6:** Total cumulative mpox cases computed from the scenario in Fig. 13 (Contacts reduction, while fixing adults and children vaccination). Here, $t_{w}$ represents the intervention week.

Intensity of intervention	$t_{w}$:10	$t_{w}$:20	$t_{w}$:30	$t_{w}$:37.81
0%	3333	6648	12 985	21 720
20%	3251	6488	12 677	21 208
40%	3176	6341	12 394	20 738
60%	3107	6206	12 134	20 306
80%	3043	6082	11 896	19 909

**Table S.7:** Total cumulative mpox cases computed from the scenario in Fig. 14 (Combined contacts reduction and vaccination increase). Here, $t_{w}$ represents the intervention week.

Intensity of intervention	$t_{w}$:10	$t_{w}$:20	$t_{w}$:30	$t_{w}$:37.81
0%	3333	6648	12 985	21 720
20%	2910	5840	11 446	19 176
40%	2629	5303	10 421	17 479
60%	2428	4919	9688	16 267
80%	2278	4631	9138	15 356

## Data Availability

The data used for this study are available in the WHO Global Mpox Trends https://worldhealthorg.shinyapps.io/mpx_global#sec-down. We collected the weekly reported Mpox cases for DRC from January 7, 2024 to April 6, 2025.

## Appendix Supplementary material

### A.1 Derivation of the control reproduction number

The matrix F, describing the new infections and matrix V describing all other transitions within the infectious stages of the model are defined as follows:

$$F=\left(\begin{array}{c} \lambda_{a}^{h}\;S_{a}^{h}\\ \lambda_{a}^{h}\;\left(1-{ɛ}_{v}\right)V_{a}^{h}\\ 0\\ 0\\ \lambda_{a}^{l}\;S_{a}^{l}\\ 0\\ 0\\ 0\\ \lambda_{c}\;S_{c}\\ \lambda_{c}\;\left(1-{ɛ}_{v}\right)V_{c}\\ 0\\ 0 \end{array}\right),\;\;V=\left(\begin{array}{c} \left(\sigma_{a}^{h}+\theta_{a}^{h}\right)E_{a}^{h}\\ \sigma_{a}^{hv}E_{a}^{hv}-\theta_{a}^{h}E_{a}^{h}\\ \left(\delta_{a}^{h}+\gamma_{a}^{h}\right)\;I_{a}^{h}-\sigma_{a}^{h}\;E_{a}^{h}\\ \left(\delta_{a}^{hv}+\gamma_{a}^{hv}\right)\;I_{a}^{hv}-\sigma_{a}^{hv}\;E_{a}^{hv}\\ \left(\sigma_{a}^{l}+\theta_{a}^{l}\right)E_{a}^{l}\\ \sigma_{a}^{lv}E_{a}^{lv}-\theta_{a}^{l}E_{a}^{l}\\ \left(\delta_{a}^{l}+\gamma_{a}^{l}\right)\;I_{a}^{l}-\sigma_{a}^{l}\;E_{a}^{l}\\ \left(\delta_{a}^{lv}+\gamma_{a}^{lv}\right)\;I_{a}^{lv}-\sigma_{a}^{lv}\;E_{a}^{lv}\\ \left(\sigma_{c}+\theta_{c}\right)E_{c},\\ \sigma_{c}^{v}E_{c}^{v}-\theta_{c}E_{c}\\ \left(\delta_{c}+\gamma_{c}\right)\;I_{c}-\sigma_{c}\;E_{c}\\ \left(\delta_{c}^{v}+\gamma_{c}^{v}\right)\;I_{c}^{v}-\sigma_{c}^{v}\;E_{c}^{v} \end{array}\right)$$

Taking the partial derivatives of F and V with respect to the disease classes $E_{a}^{h}$, $E_{a}^{hv}$, $I_{a}^{h}$, $I_{a}^{hv}$, $E_{a}^{l}$, $E_{a}^{lv}$, $I_{a}^{l}$, $I_{a}^{lv}$, $E_{c}$, $E_{c}^{v}$, $I_{c}$, $I_{c}^{v}$ and evaluating at the disease free equilibrium $\nu_{0}=\left(S_{a}^{h*},V_{a}^{h*},0,0,0,0,0,S_{a}^{l*},0,0,0,0,0,0,\right)\left(S_{c}^{*},V_{c}^{*},0,0,0,0,0\right)$ we have (see Box I):

The control reproduction number for the whole population is given by the spectral radius of $FV^{-1}$, that is, $R_{0}^{c}=\rho \left(FV^{-1}\right)$, where

(A.1) $$R_{0}^{c}=\frac{\left(R_{aa}+R_{cc}\right)+\sqrt{{\left(R_{aa}+R_{cc}\right)}^{2}-4\left(R_{aa}R_{cc}-R_{ac}R_{ca}\right)}}{2}.$$

Box I

$$F=\left(\begin{array}{cccccccccccc} 0 & 0 & \frac{\left(\beta_{a}^{s}+\beta_{a}^{hh}\right)S_{a}^{h*}}{N_{a}^{h*}} & \frac{\varphi \left(\beta_{a}^{s}+\beta_{a}^{hh}\right)S_{a}^{h*}}{N_{a}^{h*}} & 0 & 0 & \frac{\beta_{a}^{hl}S_{a}^{h*}}{N_{a}^{l*}} & \frac{\varphi \beta_{a}^{hl}S_{a}^{h*}}{N_{a}^{l*}} & 0 & 0 & \frac{\beta_{ac}^{h}S_{a}^{h*}}{N_{c}^{*}} & \frac{\varphi \beta_{ac}^{h}S_{a}^{h*}}{N_{c}^{*}}\\ 0 & 0 & \frac{\left(\beta_{a}^{s}+\beta_{a}^{hh}\right)\Psi_{v}^{h*}}{N_{a}^{h*}} & \frac{\varphi \left(\beta_{a}^{s}+\beta_{a}^{hh}\right)\Psi_{v}^{*}}{N_{a}^{h*}} & 0 & 0 & \frac{\beta_{a}^{hl}\Psi_{v}^{h*}}{N_{a}^{l*}} & \frac{\varphi \beta_{a}^{hl}\Psi_{v}^{*}}{N_{a}^{l*}} & 0 & 0 & \frac{\beta_{ac}^{h}\Psi_{v}^{h*}}{N_{c}^{*}} & \frac{\varphi \beta_{ac}^{h}\Psi_{v}^{h*}}{N_{c}^{*}}\\ 0 & 0 & 0 & 0 & 0 & 0 & 0 & 0 & 0 & 0 & 0 & 0\\ 0 & 0 & 0 & 0 & 0 & 0 & 0 & 0 & 0 & 0 & 0 & 0\\ 0 & 0 & \frac{\beta_{a}^{lh}S_{a}^{l*}}{N_{a}^{h*}} & \frac{\varphi \;\beta_{a}^{lh}S_{a}^{l*}}{N_{a}^{h*}} & 0 & 0 & \frac{\beta_{a}^{ll}S_{a}^{l*}}{N_{a}^{l*}} & \frac{\varphi \;\beta_{a}^{ll}S_{a}^{l*}}{N_{a}^{l*}} & 0 & 0 & \frac{\beta_{ac}^{l}S_{a}^{l*}}{N_{c}^{*}} & \frac{\varphi \beta_{ac}^{l}S_{a}^{l*}}{N_{c}^{*}}\\ 0 & 0 & 0 & 0 & 0 & 0 & 0 & 0 & 0 & 0 & 0 & 0\\ 0 & 0 & 0 & 0 & 0 & 0 & 0 & 0 & 0 & 0 & 0 & 0\\ 0 & 0 & 0 & 0 & 0 & 0 & 0 & 0 & 0 & 0 & 0 & 0\\ 0 & 0 & \frac{\beta_{ca}S_{c}^{*}}{N_{a}^{h*}} & \frac{\varphi \beta_{ca}S_{c}^{*}}{N_{a}^{h*}} & 0 & 0 & \frac{\beta_{ca}S_{c}^{*}}{N_{a}^{l*}} & \frac{\varphi \beta_{ca}S_{c}^{*}}{N_{a}^{l*}} & 0 & 0 & \frac{\beta_{cc}S_{c}^{*}}{N_{c}^{*}} & \frac{\varphi \;\beta_{cc}S_{c}^{*}}{N_{c}^{*}}\\ 0 & 0 & \frac{\beta_{ca}\Psi_{v}^{c*}}{N_{a}^{h*}} & \frac{\varphi \beta_{ca}\Psi_{v}^{c*}}{N_{a}^{h*}} & 0 & 0 & \frac{\beta_{ca}\Psi_{v}^{c*}}{N_{a}^{l*}} & \frac{\varphi \beta_{ca}\Psi_{v}^{c*}}{N_{a}^{l*}} & 0 & 0 & \frac{\beta_{cc}\Psi_{v}^{c*}}{N_{c}^{*}} & \frac{\varphi \;\beta_{cc}\Psi_{v}^{c*}}{N_{c}^{*}}\\ 0 & 0 & 0 & 0 & 0 & 0 & 0 & 0 & 0 & 0 & 0 & 0\\ 0 & 0 & 0 & 0 & 0 & 0 & 0 & 0 & 0 & 0 & 0 & 0 \end{array}\right)$$

where, $\Psi_{v}^{h*}=\left(1-{ɛ}_{v}\right)\;V_{a}^{h*},\;\Psi_{v}^{c*}=\left(1-{ɛ}_{v}\right)\;V_{c}^{*}$, and

$$V=\left(\begin{array}{cccccccccccc} \Phi_{1}^{h} & 0 & 0 & 0 & 0 & 0 & 0 & 0 & 0 & 0 & 0 & 0\\ -\theta_{a}^{h} & \sigma_{a}^{hv} & 0 & 0 & 0 & 0 & 0 & 0 & 0 & 0 & 0 & 0\\ -\sigma_{a}^{h} & 0 & \Phi_{2}^{h} & 0 & 0 & 0 & 0 & 0 & 0 & 0 & 0 & 0\\ 0 & -\sigma_{a}^{hv} & 0 & \Phi_{2}^{hv} & 0 & 0 & 0 & 0 & 0 & 0 & 0 & 0\\ 0 & 0 & 0 & 0 & \Phi_{1}^{l} & 0 & 0 & 0 & 0 & 0 & 0 & 0\\ 0 & 0 & 0 & 0 & -\theta_{a}^{l} & \sigma_{a}^{lv} & 0 & 0 & 0 & 0 & 0 & 0\\ 0 & 0 & 0 & 0 & -\sigma_{a}^{l} & 0 & \Phi_{2}^{l} & 0 & 0 & 0 & 0 & 0\\ 0 & 0 & 0 & 0 & 0 & -\sigma_{a}^{lv} & 0 & \Phi_{2}^{lv} & 0 & 0 & 0 & 0\\ 0 & 0 & 0 & 0 & 0 & 0 & 0 & 0 & \Phi_{1}^{c} & 0 & 0 & 0\\ 0 & 0 & 0 & 0 & 0 & 0 & 0 & 0 & -\theta_{c} & \sigma_{c}^{v} & 0 & 0\\ 0 & 0 & 0 & 0 & 0 & 0 & 0 & 0 & -\sigma_{c} & 0 & \Phi_{2}^{c} & 0\\ 0 & 0 & 0 & 0 & 0 & 0 & 0 & 0 & 0 & -\sigma_{c}^{v} & 0 & \Phi_{2}^{cv} \end{array}\right)$$

where

$$\Phi_{1}^{h}=\sigma_{a}^{h}+\theta_{a}^{h},\;\Phi_{2}^{h}=\left(\delta_{a}^{h}+\gamma_{a}^{h}\right),\;\Phi_{2}^{hv}=\left(\delta_{a}^{hv}+\gamma_{a}^{hv}\right),\;\Phi_{1}^{l}=\sigma_{a}^{l}+\theta_{a}^{l},\;\Phi_{2}^{l}=\left(\delta_{a}^{l}+\gamma_{a}^{l}\right),\;\Phi_{2}^{lv}=\left(\delta_{a}^{lv}+\gamma_{a}^{lv}\right),$$

$$\Phi_{1}^{c}=\left(\sigma_{c}+\theta_{c}\right),\;\Phi_{2}^{c}=\left(\delta_{c}+\gamma_{c}\right),\;\Phi_{2}^{cv}=\left(\delta_{c}^{v}+\gamma_{c}\right).$$

### A.2 Sensitivity analysis

In this subsection, we investigate sensitivity analysis on the parameters of the model (2.1), to determine the most influential parameters which drive the dynamics of Mpox. To achieve this, we shall use the Latin hypercube sampling (LHS) [71], [72], [73], [74], with distribution given in Table S.1. We then compute the Partial Rank Correlation Coefficient (PRCC) for each parameter involved in the sensitivity.

The results of the sensitivity analysis of the mpox control reproduction number, $R_{0}^{c}$, are presented in Fig. S.1 (left panel). The most influential parameters positively associated with $R^{c}0$ are the sexual transmission rate among adults, $\beta_{a}^{s}$ (PRCC: 0.9149); the community-based transmission rate among high-risk adults, $\beta_{a}^{hh}$ (PRCC: 0.9152); and the community-based transmission rate from infectious low-risk adults to susceptible high-risk adults, $\beta_{a}^{hl}$ (PRCC: 0.2578). Other parameters with positive correlations include $\beta_{a}^{lh}$ (PRCC: 0.4794), $\beta_{a}^{ll}$ (PRCC: 0.0133), $\beta_{ca}$ (PRCC: 0.2288), $\beta_{ac}^{h}$ (PRCC: 0.0363), $\beta_{ac}^{l}$ (PRCC: 0.0219), $\beta_{cc}$ (PRCC: 0.0978), and the proportion of sexually active adults in the high-risk group, φ (PRCC: 0.9258). Increases in any of these parameters lead to a corresponding rise in the control reproduction number, thereby elevating the potential disease burden in the population. Conversely, several parameters exhibit negative correlations with $R^{c}0$. These include mpox vaccine effectiveness, ${ɛ}_{v}$ (PRCC: −0.1589); pre-exposure vaccination rates for high-risk adults, $\alpha a^{h}$ (PRCC: −0.1716), and for children, $\alpha_{c}$ (PRCC: −0.0133); and post-exposure vaccination rates for high-risk adults, $\theta_{a}^{h}$ (PRCC: −0.7262), low-risk adults, $\theta_{a}^{l}$ (PRCC: −0.0670), and children, $\theta_{c}$ (PRCC: −0.0523). Overall, the post-exposure vaccination rate for high-risk adults emerges as the most influential negatively correlated parameter, underscoring its critical role in reducing the control reproduction number.

The results of the sensitivity analysis for the epidemic peak size of total infected individuals are presented in Fig. S.1 (right panel). With respect to the epidemic peak size, the most influential positively correlated parameters are the sexual transmission rate among high-risk adults, $\beta_{a}^{s}$ (PRCC: 0.6314); the community-based transmission rate among high-risk adults, $\beta_{a}^{hh}$ (PRCC: 0.6136); and the transmission rate from infectious high-risk adults to susceptible low-risk adults, $\beta_{a}^{lh}$ (PRCC: 0.5057). Other positively correlated parameters include $\beta_{cc}$ (PRCC: 0.1969), $\beta_{ac}^{h}$ (PRCC: 0.0872), $\beta_{ac}^{l}$ (PRCC: 0.0248), and the proportion of adults in the high-risk group, φ (PRCC: 0.8604). Increases in any of these parameters lead to a corresponding rise in the epidemic peak size. Conversely, several parameters exhibit negative correlations with the epidemic peak size. These include mpox vaccine efficacy, ${ɛ}_{v}$ (PRCC: −0.9093); pre-exposure vaccination rate among high-risk adults, $\alpha_{a}^{h}$ (PRCC: −0.6052); pre-exposure vaccination rate among children, $\alpha_{c}$ (PRCC: −0.1954); and the post-exposure vaccination rates for high-risk adults, $\theta_{a}^{h}$ (PRCC: −0.2084), and for children, $\theta_{c}$ (PRCC: −0.0141). Increasing any of these parameters reduces the epidemic peak size.

### A.3 Supplementary figures

See Fig. S.2, Fig. S.3, Fig. S.4, Fig. S.5, Fig. S.6 and Table S.2

### A.4 Supplementary tables

See Table S.3, Table S.4, Table S.5, Table S.6, Table S.7.
